# Atrial fibrillation and retinal imaging-based oculomics: A cross-sectional and longitudinal analysis of two large cohorts

**DOI:** 10.1371/journal.pdig.0001661

**Published:** 2026-09-10

**Authors:** Josef Huemer, Kelsey V. Stuart, Yukun Zhou, Dominic J. Williamson, Peter Woodward-Court, Alastair R. Mobley, Arvind Chandratheva, Florian Berger, Nay Aung, Steffen E. Petersen, UK Biobank Eye and Vision Consortium, Anthony P. Khawaja, Axel Petzold, Jugnoo Rahi, Alastair K. Denniston, Dipak Kotecha, Pearse A. Keane, Praveen J. Patel, Siegfried K. Wagner

**Affiliations:** 1 NIHR Moorfields Biomedical Research Centre, London, United Kingdom; 2 Institute of Ophthalmology, University College London, London, United Kingdom; 3 Department of Ophthalmology and Optometry, Kepler University Hospital, Linz, Austria; 4 NIHR Birmingham Biomedical Research Centre, University Hospitals Birmingham NHS Foundation Trust, Birmingham, United Kingdom; 5 Department of Cardiovascular Sciences, University of Birmingham, Birmingham, United Kingdom; 6 Department of Brain Repair and Rehabilitation, Stroke Research Centre, UCL Queen Square Institute of Neurology, London, United Kingdom; 7 The National Hospital for Neurology and Neurosurgery, UCL Institute of Neurology, London, United Kingdom; 8 Pediatric Cardiology, Pediatric Heart Center, University Children’s Hospital Zurich, Switzerland; 9 William Harvey Research Institute, NIHR Barts Biomedical Research Centre, Queen Mary University of London, London, United Kingdom; 10 Barts Heart Centre, St Bartholomew’s Hospital, Barts Health NHS Trust, London, United Kingdom; 11 MRC Epidemiology Unit, University of Cambridge, Cambridge, United Kingdom; 12 Rothschild Foundation Hospital, Paris, France; 13 Department of Ophthalmology and Visual Sciences, The Chinese University of Hong Kong; 14 UCL Great Ormond Street Institute of Child Health, London, United Kingdom; 15 Great Ormond Street Hospital NHS Foundation Trust, London, United Kingdom; 16 Ulverscroft Vision Research Group, UCL London, London, United Kingdom; 17 Julius Center, University Medical Center Utrecht, the Netherlands; Xinjiang Medical University Affiliated First Hospital, CHINA

## Abstract

We investigate the association between multimodal retinal imaging parameters with prevalent and incident atrial fibrillation (AF). For this cross-sectional and longitudinal analysis we utilized data from two independent cohorts: AlzEye (retrospective, age ≥ 40 years, 2008–2018) and UK Biobank (UKB; prospective, age 40–69 years, recruited 2006–2010). Retinal sublayer thicknesses and retinovascular indices were extracted from macular optical coherence tomography and color retinal photography, respectively. AF was ascertained via self-report and hospital admissions, association between retinal metrics with prevalent and incident AF were estimated using mixed-effects linear and frailty models. In AlzEye (n = 50,651), prevalent AF (n = 5,375; 11%) was associated with significantly smaller retinal venular calibre (-0.05 µm, 95% CI: -0.08, -0.03) and thinner macular retinal nerve fibre layer (-0.69 µm, 95% CI: -0.91, -0.46), ganglion cell-inner plexiform layer (mGCIPL; -1.96 µm, 95% CI: -2.34, -1.58), inner nuclear layer (-0.57 µm, 95% CI: -0.75, -0.38), and photoreceptor layer (-0.93 µm, 95% CI: -1.38, -0.48). Venular fractal dimension was lower (-0.08, 95% CI: -0.11, -0.06), while arteriolar fractal dimension was higher (0.05, 95% CI: 0.02, 0.08) in individuals with AF. Evidence of thinner mGCIPL was replicated in UKB (-1.19 µm, 95% CI: -1.73, -0.65). In UKB (n = 39,013 baseline without AF), 838 developed AF over a mean of 4.0 years. Adjusting for sociodemographic, clinical and lifestyle factors, each SD decrease in mGCIPL thickness was associated with a significant 27% increased hazard of AF (95% CI: 5%, 54%). Differences in the inner retinal sublayers, corresponding to retinal ganglion cells, are associated with prevalent and incident AF across two large independent cohorts, supporting the potential role of widely-accessible retinal imaging for early detection and better management of AF.

## Author summary

Atrial fibrillation, one of the most common heart conditions causing an irregular heart rhythm, affects millions of people worldwide and significantly increases the risk of stroke and heart failure. Detecting it early, before symptoms arise, remains a major clinical challenge. In this study, we explored whether the retina could offer clues about a person’s cardiac health. Because the retina shares structural and vascular similarities with the brain and heart, changes visible on routine eye scans may reflect broader changes occurring elsewhere in the body. Using eye imaging data from over 90,000 individuals across two large independent datasets, we found that people with atrial fibrillation show measurable thinning of specific inner retinal layers, the ganglion cell and inner plexiform layer, and the inner nuclear layer, compared to those without atrial fibrillation. Crucially, individuals with a thinner retina at baseline were significantly more likely to develop atrial fibrillation in the future, even before any cardiac diagnosis had been made, underlining the difficulty of establishing the diagnosis. These findings suggest that standard, non-invasive retinal scans which are widely used both in eye clinics and community settings, could potentially help identify individuals at elevated cardiac risk, potentially enabling earlier intervention and improved outcomes.

## Introduction

Atrial fibrillation (AF) is already one of the commonest cardiac conditions and expected to further double in prevalence over the next two decades [1]. It is a leading contributor to several complications including ischemic stroke, myocardial infarction, vascular dementia and mortality [2]. Diagnosis of AF relies on capturing what is often a sporadic heart rhythm abnormality on an electrocardiogram (ECG). The likelihood of AF detection depends on aligning symptoms with ECG-based monitoring, but AF episodes themselves do not directly relate to thromboembolic outcomes [2]. Rather, AF is a thrombo-inflammatory condition closely associated with underlying vascular disease. Appropriate use of established therapy, including direct oral anticoagulants and modulators of vascular disease, have the potential for substantial public health impact, if earlier diagnosis of AF can be achieved across broader communities [1].

**Fig 1:**
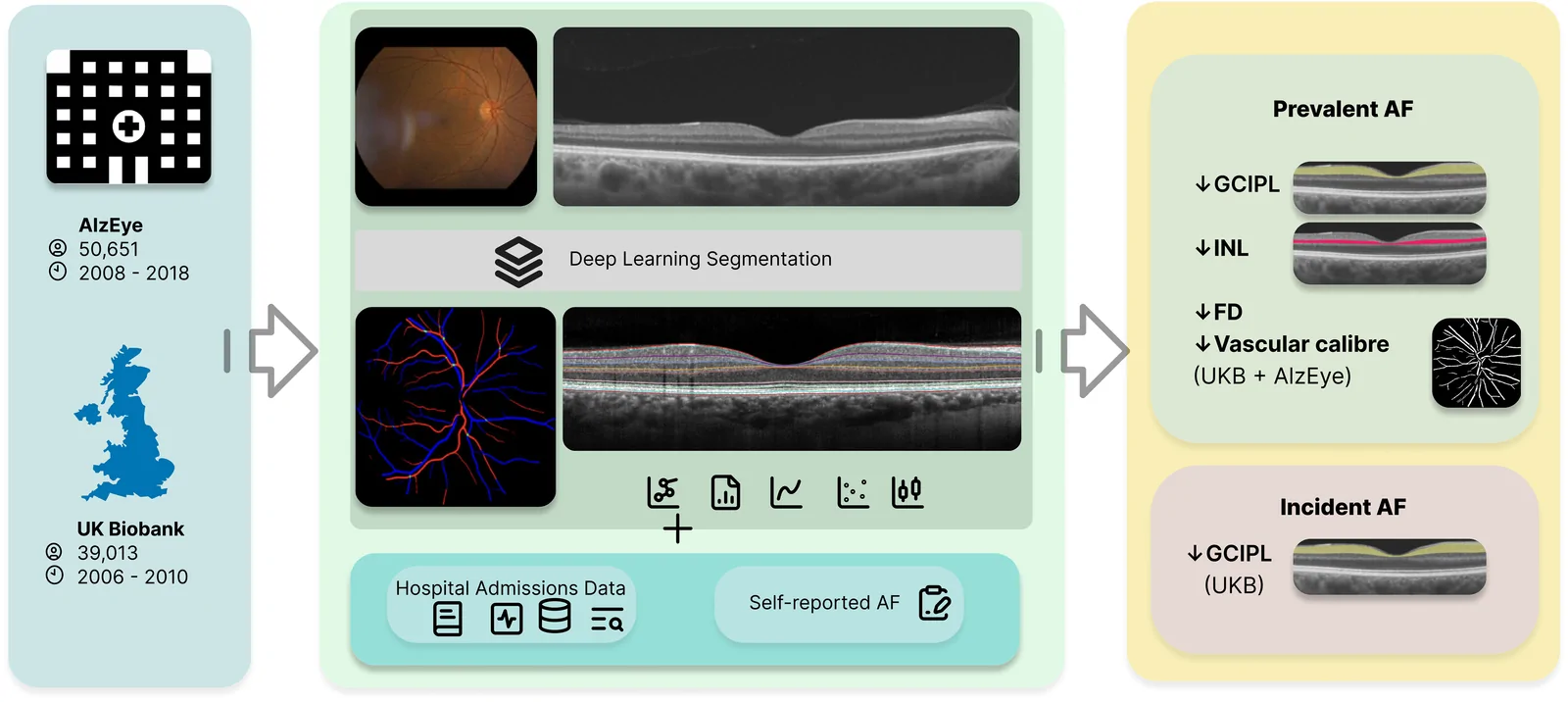
Schematic detailing study design, retinal imaging modalities, and results summary. AF: atrial fibrillation, FD: fractal dimension, GCIPL: ganglion cell-inner plexiform layer, INL: inner nuclear layer, UKB: UK Biobank.

The retina with its vasculature and multiple layers provides a non-invasive method to assess microvascular and central nervous system alterations [3]. Novel insights can be provided by analysing widely-available optical coherence tomography (OCT) scans and colour fundus photographs (CFP) using artificial intelligence (AI) assisted segmentation, classification and predictive modelling [3,4]. Retinal vasculature parameters and retinal sublayer thickness are associated with cardiovascular disease (CVD) and specifically with AF [5,6], but prior studies have been limited by size, scope and imaging modality. We hypothesised that retinovasculature and neuroretinal indices could be a biomarker of AF, providing an easily-accessible window for detection of vascular disease status in individual patients. In this study, we analysed two large independent cohorts, drawing on data from more than 90,000 individuals to understand the links between retinal morphology and the prevalence and incidence of AF (Fig 1).

## Results

**Fig 2:**
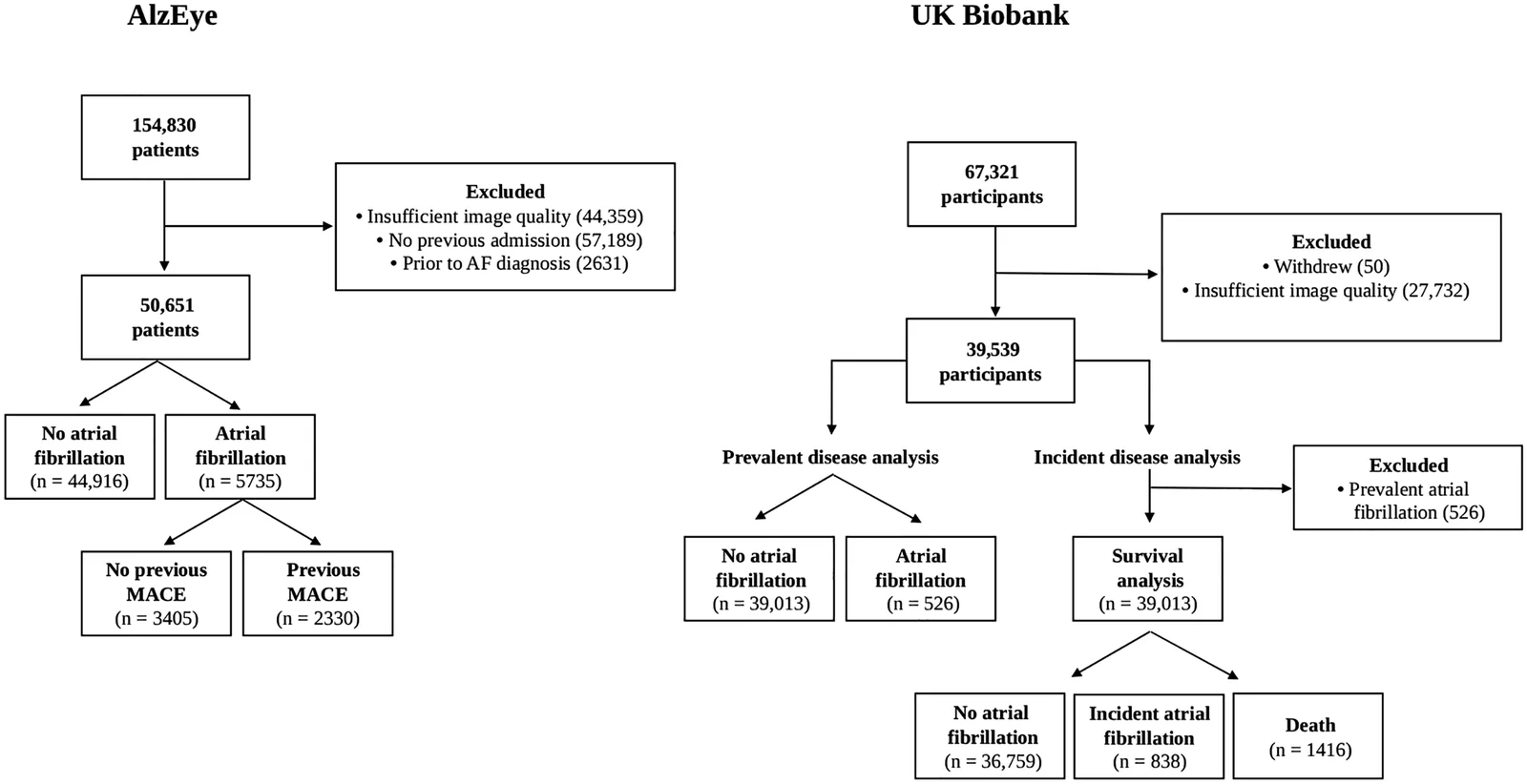
Flow chart for the AlzEye and UK Biobank cohort analyses. MACE: Major adverse cardiovascular event.

This analysis drew on data from 90,190 individuals across the two independent cohorts. The AlzEye analysis consisted of 50,651 individuals, of which 5,735 had AF (Fig 2, prevalence: 11.3%). Individuals with AF were, on average, older, more likely to be male, have hypertension and diabetes mellitus. They were also significantly more likely to have suffered a previous MACE (Table 1, 40.6% versus 10.0%, p < 0.001). The UKB analysis included 39,539 participants, of which 526 had prevalent AF at baseline (1.3%) (Fig 2). Similar to AlzEye, individuals in UKB with AF were generally older, more likely to be male, have hypertension and diabetes mellitus (Table 2). Within UKB, 10.5% of participants with AF had a previous MACE compared to 1.5% without AF (p < 0.001).

**Table 1:** Baseline characteristics of the AlzEye cohort stratified by presence of previous major adverse cardiovascular event.

			AlzEye					
			All			Without MACE		
			No AF (n = 44,916)	AF (n = 5,735)	p-value	No AF(n = 40,414)	AF (n = 3,405)	p-value
**Demographics**	Age (mean + /- SD)	Years	69.0 + /- 12.1	77.8 + /- 9.8	**<0.001**	68.8 + /- 12.1	77.3 + /- 9.9	**<0.001**
	Sex n (%)	Female	23,188 (51.6)	2756 (48.1)	**<0.001**	21,333 (92.7)	1674 (7.3)	**<0.001**
		Male	21,728 (48.4)	2979 (51.9)		19,081 (91.7)	1731 (8.3)	
	Ethnicity n (%)	South Asian	10,350 (23.0)	744 (13.0)	**<0.001**	9072 (96.4)	339 (3.6)	**<0.001**
		Black	4,595 (10.2)	264 (4.6)		4192 (96.7)	141 (3.3)	
		Other/Mixed	7157 (15.9)	840 (14.6)		6452 (93.0)	489 (7.0)	
		White	15,145 (33.7)	3001 (52.3)		13,825 (88.1)	1875 (11.9)	
		Unknown	7,669 (17.1)	886 (15.4)		6873 (92.5)	561 (7.5)	
**Clinical** **n (%)**	Hypertension	Present	36,483 (81.2)	5039 (87.9)	**<0.001**	32,369 (92.0)	2829 (8.0)	**<0.001**
	Diabetes mellitus	Present	23,112 (51.5)	2389 (41.7)	**<0.001**	20,253 (94.6)	1157 (5.4)	**<0.001**
	Previous MACE	Present	4,502 (10.0)	2330 (40.6)	**<0.001**	–		
**CFP (units)**	Arteriolar calibre (μm)	(mean + /- SD)	64.3 + /- 8.0	63.8 + /- 8.3	**<0.001**	64.3 + /- 8.0	63.5 + /- 8.3	**<0.001**
	Arteriolar distance tortuosity	(mean + /- SD)	5.67 + /- 3.66	6.01 + /- 3.93	**<0.001**	5.68 + /- 3.64	6.05 + /- 3.83	**<0.001**
	Arteriolar fractal dimension	(mean + /- SD)	1.26 + /- 0.05	1.25 + /- 0.05	**<0.001**	1.26 + /- 0.05	1.25 + /- 0.04	**<0.001**
	Venular calibre (μm)	(mean + /- SD)	72.3 + /- 8.8	71.8 + /- 9.5	**<0.001**	72.2 + /- 8.8	71.5 + /- 9.3	**<0.001**
	Venular distance tortuosity	(mean + /- SD)	3.48 + /- 1.73	3.59 + /- 1.89	**0.01**	3.47 + /- 1.72	3.58 + /- 1.84	**<0.001**
	Venular fractal dimension (d)	(mean + /- SD)	1.30 + /- 0.06	1.28 + /- 0.06	**<0.001**	1.30 + /- 0.06	1.28 + /- 0..05	**<0.001**
**Optic nerve** **(mm)**	Cup height	(mean + /- SD)	63.2 + /- 16.4	61.0 + /- 17.8	**<0.001**	63.1 + /- 16.4	60.4 + /- 18.1	**<0.001**
	Cup width	(mean + /- SD)	57.6 + /- 14.9	55.7 + /- 16.3	**<0.001**	57.5 + /- 15.0	55.3 + /- 16.5	**<0.001**
	Disc height	(mean + /- SD)	138.1 + /- 21.0	133.8 + /- 24.2	**<0.001**	138.1 + /- 21.0	133.0 + /- 24.8	**<0.001**
	Disc width	(mean + /- SD)	127.0 + /- 16.6	125.3 + /- 19.2	**<0.001**	126.9 + /- 16.7	125.0 + /- 19.7	**<0.001**
**OCT (μm)**	mRNFL	(mean + /- SD)	25.2 + /- 8.2	24.5 + /- 8.2	**<0.001**	25.2 + /- 8.1	24.6 + /- 8.0	**<0.001**
	mGCIPL	(mean + /- SD)	81.3 + /- 14.0	77.1 + /- 14.8	**<0.001**	81.6 + /- 13.9	78.0 + /- 14.5	**<0.001**
	mINL	(mean + /- SD)	41.0 + /- 6.6	40.3 + /- 6.8	**<0.001**	41.0 + /- 6.6	40.5 + /- 6.7	**<0.001**
	PRL	(mean + /- SD)	156/0 + /- 16.0	153.9 + /- 17.4	**<0.001**	156.2 + /- 15.8	155.0 + /- 16.9	**0.006**
	RPE-BM	(mean + /- SD)	26.9 + /- 8.3	28.5 + /- 10.0	**<0.001**	26.9 + /- 8.4	28.8 + /- 10.4	**<0.001**

CFP: color fundus photography, GC-IPL: ganglion cell-inner plexiform layer, INL: inner nuclear layer, MACE: major adverse cardiovascular event, mRNFL: macular retinal nerve fibre layer, OCT: optical coherence tomography, PRL: photoreceptor layer, RPE-BM: retinal pigment epithelium-Bruch’s membrane layer, SD: standard deviation.

**Table 2:** Baseline characteristics of the UK Biobank.

			UK Biobank		
			No Prevalent AF (n = 39,013)	Prevalent AF (n = 526)	p-value
**Demographics**	Age (mean + /- SD)	Years	55.4 + /- 8.2	61.5 + /- 6.4	**<0.001**
	Sex n (%)	Female	21,499 (55.1)	163 (31.0)	**<0.001**
		Male	17,514 (44.9)	363 (69.0)	
	Ethnicity n (%)	Non-White	3166 (8.1)	14 (2.7)	**<0.001**
		White	35,592 (91.2)	511 (97.1)	
		Unknown	255 (0.7)	1 (0.2)	
**Exposures** **n (%)**	Hypertension	Absent	29,789 (76.4)	280 (53.2)	**<0.001**
		Present	9224 (23.6)	246 (46.8)	
	Diabetes mellitus	Absent	37,344 (95.7)	479 (91.1)	**<0.001**
		Present	1669 (4.3)	47 (8.9)	
	Alcohol	Never	1765 (4.5)	14 (2.7)	0.11
		Previous	1311 (3.4)	20 (3.8)	
		Current	35,800 (92.1)	491 (93.5)	
	Smoking	Never	22,016 (56.7)	251 (48.1)	**<0.001**
		Previous	13,063 (33.7)	237 (45.4)	
		Current	3718 (9.6)	34 (6.5)	
	Previous MACE	Absent	38,419 (98.5)	471 (89.5)	**<0.001**
		Present	594 (1.5)	55 (10.5)	
	Incident AF	Present	838	0	NA
**CFP (units)**	Arteriolar calibre	(mean + /- SD)	61.6 + /- 5.6	61.5 + /- 5.8	0.48
	Arteriolar distance tortuosity	(mean + /- SD)	5.47 + /- 3.12	5.50 + /- 3.13	0.72
	Arteriolar fractal dimension	(mean + /- SD)	1.29 + /- 0.04	1.28 + /- 0.04	**<0.001**
	Venular calibre	(mean + /- SD)	68.0 + /- 6.7	68.7 + /- 6.9	0.013
	Venular distance tortuosity	(mean + /- SD)	3.48 + /- 1.65	3.46 + /- 1.69	0.32
	Venular fractal dimension	(mean + /- SD)	1.31 + /- 0.04	1.30 + /- 0.04	**<0.001**
**Optic nerve** **(mm)**	Cup height	(mean + /- SD)	63.2 + /- 14.8	63.6 + /- 16.9	0.96
	Cup width	(mean + /- SD)	60.8 + /- 14.4	61.8 + /- 17.7	0.58
	Disc height	(mean + /- SD)	132.6 + /- 18.1	132.3 + /- 20.1	0.55
	Disc width	(mean + /- SD)	125.4 + /- 16.6	127.1 + /- 20.2	0.07
**OCT (μm)**	mRNFL	(mean + /- SD)	32.2 + /- 3.5	31.7 + /- 3.7	**<0.001**
	mGCIPL	(mean + /- SD)	76.1 + /- 6.3	73.9 + /- 7.1	**<0.001**
	mINL	(mean + /- SD)	35.8 + /- 2.6	35.7 + /- 2.6	0.64
	PRL	(mean + /- SD)	165.1 + /- 9.1	165.0 + /- 9.1	0.84
	RPE-BM	(mean + /- SD)	22.9 + /- 3.9	23.2 + /- 2.6	**<0.001**

AF: atrial fibrillation, CFP: color fundus photography, GC-IPL: ganglion cell-inner plexiform layer, INL: inner nuclear layer, MACE: major adverse cardiovascular event, mRNFL: macular retinal nerve fibre layer, OCT: optical coherence tomography, PRL: photoreceptor layer, RPE-BM: retinal pigment epithelium-Bruch’s membrane layer, SD: standard deviation. *Numbers suppressed due to small size.

### Prevalent atrial fibrillation

In AlzEye, after adjustment for age, sex, ethnicity, hypertension and diabetes mellitus, individuals with prevalent AF had significantly thinner retinal venular calibre compared to individuals without AF (-0.05, 95% CI: -0.08, -0.03, p = 9.1 x 10^-5^). Retinal venular fractal dimension was significantly lower in those with AF (-0.08, 95% CI: -0.11, -0.06, p = 1.1 x 10^-10^), whereas arteriolar fractal dimension was higher (0.05, 95% CI: 0.02, 0.08, p = 1.7 x 10^-4^). Significantly thinner retinal sublayers in the mRNFL (-0.69 microns, 95% CI: -0.91, -0.46, p = 1.9 x 10^-9^), mGCIPL (-1.96 microns, 95% CI: -2.34, -1.58, p < 2.0 x 10^-16^), mINL (-0.57 microns, 95% CI: -0.75, -0.38, p = 1.4 x 10^-9^) and PRL (-0.93 microns, 95% CI: -1.38, -0.48, p = 5,1x10^-5^) were seen among those with prevalent AF. Given the high prevalence of MACE in individuals with AF, we restricted the analysis to those with no previous MACE. Similar changes were seen in venular calibre and venular fractal dimension, mRNFL, mGCIPL and INL.

**Fig 3:**
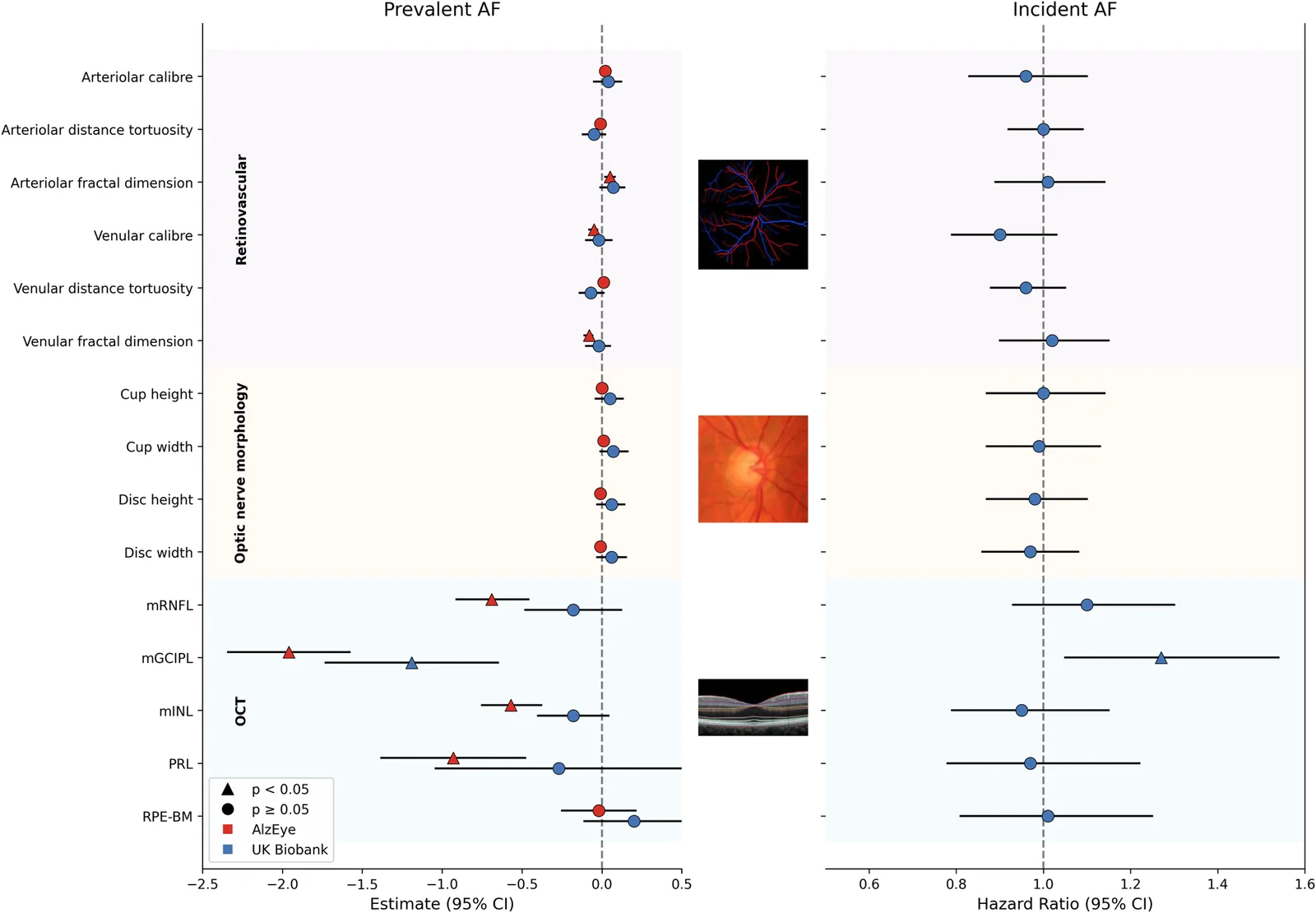
Summary of regression coefficients for prevalent and incident atrial fibrillation (AF) in both the AlzEye and UK Biobank cohorts. For prevalent AF, coefficients are derived from adjusted mixed effects linear regression. For incident atrial fibrillation, hazard ratios were derived from adjusted frailty models per standard deviation decrease. AF: atrial fibrillation, CI: confidence interval, HR: hazard ratio, mGC-IPL: macular ganglion cell-inner plexiform layer, mINL: macular inner nuclear layer, mRNFL: macular retinal nerve fibre layer, OCT: optical coherence tomography, PRL: photoreceptor layer, RPE-BM: retinal pigment epithelium-Bruch’s membrane layer.

Replicating the analysis in the UKB cohort of 526 individuals with prevalent AF using the same variable adjustment (S1 Table) and additionally adjusting for alcohol consumption and smoking status, effect estimates were of the same direction as AlzEye, with smaller magnitudes of difference (Table 3). In UKB, only thinner mGCIPL was significantly different in those with AF (-1.19 microns, 95% CI: -1.73, -0.65, p = 1.5 x 10^-5^, Fig 3). Sensitivity analysis excluding those with previous MACE in UKB did not result in any significant change in effect estimates. Individuals with prevalent AF had significantly thinner mGCIPL (-1.15 microns Table 4, 95% CI: -1.72, -0.58, n prevalent AF = 473, S2 Table). Similarly, excluding those with self-reported eye diseases in UKB did not alter the direction or magnitude of significant effect estimates (S3 Table).

**Table 3:** Association between prevalent atrial fibrillation and retinal morphology. Estimates are derived from multivariable mixed effects linear regression adjusting for age, sex, ethnicity, hypertension and diabetes mellitus; in the UK Biobank analysis, models were also adjusted for alcohol consumption and smoking status.

Prevalent Atrial Fibrillation		AlzEye – all (n AF = 5735)		AlzEye – No MACE (n AF = 3405)		UK Biobank (n AF = 526)	
Characteristic		Estimate (95% CI)	*p-*value	Estimate (95% CI)	*p*-value	Estimate (95% CI)	*p*-value
**Retinovascular** **(per SD)**	Arteriolar calibre	0.02 (-0.01, 0.04)	0.20	0.01 (-0.02, 0.04)	0.54	0.04 (-0.05, 0.12)	0.40
	Arteriolar distance tortuosity	-0.01 (-0.03, 0.02)	0.59	0.00 (-0.03, 0.03)	0.85	-0.05 (-0.12, 0.02)	0.19
	Arteriolar fractal dimension	0.05 (0.02, 0.08)	**1.7 x 10** ^**–4**^	0.05 (0.02, 0.08)	**0.004**	0.07 (-0.01, 0.14)	0.09
	Venular calibre	-0.05 (-0.08, -0.03)	**9.1 x 10** ^**–5**^	-0.06 (-0.09, -0.03)	**2.7 x 10** ^**–4**^	-0.02 (-0.10, 0.06)	0.57
	Venular distance tortuosity	0.01 (-0.02, 0.03)	0.66	0.00 (-0.03, 0.04)	0.76	-0.07 (-0.14, 0.01)	0.07
	Venular fractal dimension	-0.08 (-0.11, -0.06)	**1.1 x 10** ^**–10**^	-0.08 (-0.11, -0.05)	**9.5 x 10** ^**–7**^	-0.02 (-0.10, 0.05)	0.53
**Optic nerve morphology** **(per SD)**	Cup height	0.00 (-0.03, 0.02)	0.77	-0.01 (-0.05, 0.03)	0.60	0.05 (-0.04, 0.13)	0.25
	Cup width	0.01 (-0.02, 0.03)	0.68	0.01 (-0.03, 0.05)	0.63	0.07 (-0.01, 0.16)	0.09
	Disc height	-0.01 (-0.04, 0.02)	0.48	-0.02 (-0.06, 0.01)	0.23	0.06 (-0.03, 0.14)	0.17
	Disc width	-0.01 (-0.04, 0.02)	0.49	-0.01 (-0.04, 0.03)	0.63	0.06 (-0.03, 0.15)	0.17
**OCT** **(μm)**	mRNFL	-0.69 (-0.91, -0.46)	**1.9 x 10** ^**–9**^	-0.68 (-0.96, -0.40)	**2.1 x 10** ^**–6**^	-0.18 (-0.48, 0.12)	0.23
	mGCIPL	-1.96 (-2.34, -1.58)	**<2.0 x 10** ^**–16**^	-1.59 (-2.06, -1.11)	**5.5 x 10** ^**–11**^	-1.19 (-1.73, -0.65)	**1.5 x 10** ^**–5**^
	mINL	-0.57 (-0.75, -0.38)	**1.4 x 10** ^**–9**^	-0.50 (-0.73, -0.27)	**2.4 x 10** ^**–5**^	-0.18 (-0.40, 0.04)	0.11
	PRL	-0.93 (-1.38, -0.48)	**5.1 x 10** ^**–5**^	-0.29 (-0.85, 0.26)	0.30	-0.27 (-1.04, 0.50)	0.50
	RPE-BM	-0.02 (-0.25, 0.21)	0.88	0.16 (-0.13, 0.45)	0.27	0.20 (-0.11, 0.53)	0.20

CI: confidence interval, GC-IPL: ganglion cell-inner plexiform layer, INL: inner nuclear layer, MACE: major adverse cardiovascular event, mRNFL: macular retinal nerve fibre layer, OCT: optical coherence tomography, PRL: photoreceptor layer, RPE-BM: retinal pigment epithelium-Bruch’s membrane layer, SD: standard deviation. 3.

**Table 4:** Association between incident atrial fibrillation and retinal morphology. Estimates are derived from frailty models. Model 1 was unadjusted; Model 2 included adjustment for age, sex and ethnicity. Model 3 additionally included adjustment for hypertension, diabetes mellitus, alcohol consumption, and smoking status.

Incident Atrial Fibrillation		Model 1		Model 2		Model 3	
		HR (95% CI)	*p* value	HR (95% CI)	*p* value	HR (95% CI)	*p* value
**Retinovascular indices** (per SD increase)	Arteriolar calibre	0.92 (0.81, 1.05)	0.23	0.96 (0.83, 1.11)	0.59	0.96 (0.83, 1.10)	0.52
	Arteriolar distance tortuosity	1.02 (0.94, 1.11)	0.63	1.00 (0.92, 1.09)	0.95	1.00 (0.92, 1.09)	>0.99
	Arteriolar fractal dimension	0.87 (0.77, 0.98)	**0.020**	1.00 (0.88, 1.13)	0.95	1.01 (0.89, 1.14)	0.93
	Venular calibre	1.02 (0.90, 1.16)	0.73	0.92 (0.80, 1.05)	0.21	0.90 (0.79, 1.03)	0.14
	Venular distance tortuosity	1.00 (0.91, 1.09)	0.94	0.96 (0.88, 1.05)	0.33	0.96 (0.88, 1.05)	0.33
	Venular fractal dimension	0.86 (0.76, 0.97)	**0.017**	1.02 (0.90, 1.15)	0.80	1.02 (0.90, 1.15)	0.80
**Optic nerve morphology** (per SD increase)	Cup height	1.03 (0.90, 1.17)	0.69	0.98 (0.86, 1.12)	0.79	1.00 (0.87, 1.14)	0.95
	Cup width	1.04 (0.92, 1.17)	0.56	0.97 (0.86, 1.11)	0.69	0.99 (0.87, 1.13)	0.89
	Disc height	1.01 (0.89, 1.13)	0.92	0.99 (0.87, 1.12)	0.84	0.98 (0.87, 1.10)	0.72
	Disc width	1.01 (0.90, 1.12)	0.89	0.97 (0.86, 1.09)	0.60	0.97 (0.86, 1.08)	0.55
**OCT** (per SD decrease)	mRNFL	1.18 (1.01, 1.37)	**0.039**	1.08 (0.91, 1.27)	0.38	1.10 (0.93, 1.30)	0.28
	mGCIPL	1.56 (1.32, 1.85)	**3.4 x 10** ^**–7**^	1.28 (1.05, 1.54)	**0.012**	1.27 (1.05, 1.54)	**0.014**
	mINL	0.90 (0.75, 1.09)	0.28	0.96 (0.79, 1.16)	0.67	0.95 (0.79, 1.15)	0.62
	PRL	0.99 (0.83, 1.19)	0.94	1.05 (0.85, 1.32)	0.64	0.97 (0.78, 1.22)	0.82
	RPE-BM	0.99 (0.81, 1.22)	0.96	1.05 (0.83, 1.32)	0.66	1.01 (0.81, 1.25)	0.93

CI: confidence interval, GC-IPL: ganglion cell-inner plexiform layer, HR: hazard ratio, INL: inner nuclear layer, MACE: major adverse cardiovascular event, mRNFL: macular retinal nerve fibre layer, OCT: optical coherence tomography, PRL: photoreceptor layer, RPE-BM: retinal pigment epithelium-Bruch’s membrane layer, SD: standard deviation.

### Incident atrial fibrillation

After excluding those with prevalent AF in the UKB cohort, 39,013 individuals (total person-years follow-up: 402,815.6 years, mean follow up: 10.3 + /- 1.4 years) were analyzed of which 838 (2.1%) developed AF during the study period at a mean time of 3.95 + /- 1.91 years from baseline assessment (Fig 2). Lower arteriolar fractal dimension (HR: 1.15 per SD decrease, 95% CI: 1.02, 1.30, p = 0.02) and venular fractal dimension (HR 1.16, 95% CI: 1.03, 1.32, p = 0.017) were associated with greater risk of incident AF. Individuals with thinner mRNFL (HR: 1.18 per SD decrease, 95% CI: 1.01, 1.37) and mGCIPL (HR: 1.56 per SD decrease, 95% CI: 1.32, 1.85) had an increased risk of incident AF. On fully adjusted models, only the mGCIPL remained significantly associated with AF incidence with every SD decrease in mGCIPL associated with 27% greater risk of AF (HR: 1.27, 95% CI: 1.05, 1.54, p = 0.014, Fig 3).

## Discussion

Drawing on data across two large distinct cohorts, we observed differences in retinal sublayer thickness and retinal vasculature in individuals with AF. Prevalent AF was consistently associated with a thinner mGCIPL. In the hospital-attending cohort of AlzEye, even after restricting to those without concurrent MACE, we found several differences in retinovascular indices as well as mINL thickness. Differences in the mGCIPL thickness were also evident in individuals with incident AF from the UKB longitudinal cohort. Our report, which represents to our knowledge by some magnitude the largest reported analysis of retinal morphology in people with AF, underlines the mechanistic associations of AF and structural retinal changes and, once validated by further studies could support potential prognostic utility of retinal imaging in cardiac arrhythmias.

Across both our datasets we observed a thinner mGCIPL in patients with AF. This is the final output layer of the vertebrate retina, and we demonstrate an effect size that is comparable to six years older age in the UKB analysis [7]. We hypothesize that this difference is driven predominantly through retrograde transsynaptic degeneration in those with AF, which mechanistically accounts for the mGCIPL thinning seen in individuals with multiple sclerosis and, more pertinently, following ischaemic stroke [8,9]. Patients with AF have an increased risk of silent cerebral infarction, with one European prospective cohort study (Swiss-AF) [10] of 1227 AF patients demonstrating new cerebral infarctions in 5.5% of patients within 2 years. Of note, the majority were clinically silent, which may rationalize our persistent finding of thinner mGCIPL even when excluding those with documented previous myocardial and cerebrovascular events. While the mGCIPL changes could potentially be explained with appropriate neuroimaging, we could not include such analysis in our study. Swept-source OCT and OCT angiography have been used to characterize the superficial capillary plexus of the retinal macular area in AF, showing reduced retinal capillary densities and perfusion as well as thinner mGCIPL and RNFL thickness when compared to age- and sex matched controls [6,10]. Furthermore, they also show a dependence of the retinal vascular parameters with echocardiographic measurements of the left atrium. The mGCIPL changes as shown in both of our cohorts are in line with a recently published study exploring the influence of AF on the progression of glaucoma, a neurodegenerative disease [5]. The particular sensitivity of the mGCIPL to systemic pathophysiological processes is further supported by an analysis of over 42,000 UK Biobank participants that GCIPL explains approximately double the variance attributable to systemic factors, thus being the superior inner retinal biomarker [7]. To contextualise these effect sizes, the -1.96 µm mGCIPL thinning observed in the AlzEye cohort represents 37.8% of the mGCIPL standard deviation and is equivalent to approximately 13 years of age-related thinning, and approximately 4 times the mGCIPL thinning associated with daily alcohol consumption. The -1.19 µm difference in the UKB cohort similarly represents 22.9% of the standard deviation and is equivalent to approximately 8 years of aging. The combination of retrograde transsynaptic neurodegeneration from AF-associated cerebrovascular injury and direct ischaemic susceptibility of the high-metabolic-demand ganglion cell layer provides a dual mechanistic basis for the mGCIPL as a hypothetically plausible and reproducible retinal correlate of AF. Given the associations between thinner mGCIPL and AF, future research could validate our results by linking the retinal anatomic changes with neuroimaging to establish this relationship between silent cerebral infarcts, AF and retinal sublayer differences.

In the AlzEye dataset we found significantly thinner mINL in patients with AF, even those without MACE. The INL, a retinal sublayer containing the cell bodies of horizontal cells, bipolar cells, amacrine cells and interplexiform neurons is typically preserved in individuals with neurological diseases, such as Alzheimer disease and multiple sclerosis, a finding which has been attributed to the protective synaptic barrier for retrograde transsynaptic degeneration [11,12]. Our observation may instead be explained through a microvascular pathogenesis within the inner retina. The INL receives its blood supply through the deep capillary plexus of the macular vasculature, a watershed zone susceptible to subclinical ischemia in the form of retinal ischaemic perivascular lesions. These focal thinnings of the mINL have been increasingly reported with observations in participants with coexisting cardiovascular disease [13], single subcortical infarcts [14], carotid artery disease [14,15], and AF [16]. Novel high resolution OCTs in individuals affected by AF would improve visualisation of the INL morphology and characterize microvascular dysfunction of the inner retina.

The analysis of retinovascular fractal dimensions and vascular density reveal significant alterations in patients with conditions such as minor stroke, congestive heart failure, certain retinal vascular diseases, type 2 diabetes, sleep apnea, and macrovascular cardiac disease, positioning these metrics as potential biomarkers for population screening [17–20]. In our study, the AlzEye cohort demonstrated lower venular fractal dimension and, to a lesser extent, greater arteriolar fractal dimension in individuals with prevalent AF, even after MACE adjustment. Furthermore, lower arteriolar and venular fractal dimension were observed in the AlzEye group with incident AF. However, similar to the previously noted INL thinning, these distinct findings could not be replicated in the UKB cohort. This discrepancy may stem from differences in sample size, inherent population variance, and cohort characteristics, as UKB participants are generally healthier than the UK population [21], whereas the AlzEye dataset comprises hospital patients with a higher burden of systemic and ocular diseases [22]. Consequently, further investigations leveraging longitudinal data, ideally capturing progression from paroxysmal to persistent AF, are essential to clarify these associations.

While the observed retinovascular alterations and retinal sublayer thickness changes are consistent with our pathomechanistic hypothesis of a clinical association with AF, their context is paramount. The diagnosis of AF currently relies on cardiac monitoring, clinical presentation, and cardiovascular risk profiling. The limitations of ECG monitoring over a prolonged period is complicated by the fact that photoplethysmography-based monitoring is constrained by evidence quality and the requirement for physician ECG confirmation before diagnosis [1]. Retinal imaging in comparison, captures structural end-organ sequelae of AF that persist independently of arrhythmia burden at any single timepoint. We propose that these retinal findings are not a substitute for this paradigm, but rather a complementary tool. From an oculomic perspective, they highlight the complex systemic nature of AF and, if confirmed in subsequent studies assessing their predictive potential, could guide clinicians to pursue more extensive cardiac investigations, such as implantable loop recorders, in clinically equivocal cases.

Notwithstanding strengths of our report including the large sample size across two cohorts, multimodal retinal imaging, and ethnic diversity of the AlzEye cohort, there are limitations to highlight. We relied on self-report and/or hospital admissions data for identifying individuals with AF, which may be prone to misclassification bias. A 2024 manual validation study however showed high PPV for an ICD-10 code for AF (PPV 99.1%) but more modest sensitivity (77.5%) [23], echoing findings from a 2013 US-based systematic review across 16 studies (median PPV, 89%, sensitivity 79%) [24]. Because paroxysmal and asymptomatic AF will likely not be included in hospital coding or self-reports, some individuals classified as controls, and in the incident analysis, may in fact have had undiagnosed AF at the time of retinal imaging. Thus, while we can be confident of our cases, controls may be affected by AF (even more so given the diagnostic challenge in identifying asymptomatic AF) suggesting our effect measures to underestimate the true association.Thus, while we can be confident of our cases, controls may be affected by AF (even more so given the diagnostic challenge in identifying asymptomatic AF) suggesting our effect measures to underestimate the true association. While we sought to replicate our findings across two large but disparate cohorts, neither is truly representative of the broader population. Participants of UKB engage in fewer adverse health behaviour and have greater life expectancy than the general UK population [21]. In contrast, the hospital cohort of AlzEye represents one of the most ethnically diverse cohorts in the Western Hemisphere and is skewed towards those with greater levels of socioeconomic deprivation [22]. Replication however of our findings across both cohorts bolsters the argument of a true association. In the incident AF analysis, approximately 20% of individuals developed AF within 24 months of retinal imaging. Given that AF is typically paroxysmal in its early stages, our findings may be more applicable to those with prevalent rather than incident AF. Finally, as with other observational health data analyses, there may be factors which we could not account for but may plausibly influence both AF risk and retinal structure (residual confounding).

This study demonstrates that individuals with prevalent and incident AF exhibit thinner mGCIPL and mINL, alongside differences in vascular indices, with a similar but less consistent pattern observed for incident AF. The observed mGCIPL thinning may result from microvascular events associated with AF that affect the visual pathways and retina. In contrast, the novel finding of mINL thinning could be interpreted in the context of underlying subclinical ischemia reflecting systemic cardiovascular compromise. These findings, once validated in subsequent studies, highlight the potential for non-invasive retinal imaging to provide early detection and risk stratification of AF, as well as an easily-accessible opportunity to understand AF pathophysiology and directly assess end-organ damage. Given the widespread availability of retinal imaging in primary care optometrist stores across developed nations, further attention is warranted to explore the role of retina imaging for earlier diagnosis of AF, and the opportunity to target therapy addressing underlying vascular disease to reduce the large patient and healthcare burdens associated with AF.

## Materials and methods

### Design, participants and setting

This analysis included cross-sectional and longitudinal data from two separate studies – AlzEye and UK Biobank (UKB). AlzEye is a retrospective cohort study, where individual-level ophthalmic data has been linked with hospital admissions across England for 353,157 participants (154,830 have had retinal imaging). Participants were aged 40 years and over and had attended Moorfields Eye Hospital NHS Foundation Trust (MEH) in London, United Kingdom (UK) between January 1st 2008 and April 1st 2018 [22]. UK Biobank is a prospective multicenter population-based cohort study of >500,000 individuals residing in the UK aged 40–69 years. Recruitment occurred between 2006 and 2010 across 22 assessment centres. In addition to baseline questionnaires and physical measurements, a subset of 67,321 unique UKB participants additionally underwent a detailed ophthalmic assessment, including retinal imaging, either at their initial assessment visit or at the repeat imaging visit [25]. Protocols for UKB are available online (http://www.ukbiobank.ac.uk/resources/).

The AlzEye study has received institutional and ethical review board approval (REC reference: 18/LO/1163), with individual participant consent waived under an approved exemption; access to confidential patient information was additionally authorised under Section 251 of the NHS Act 2006 (CAG reference: 18/CAG/0111) [22]. UKB is conducted under the approval of the North-West Research Ethics Committee (ref: 06/MRE08/75); specific approval was obtained for this project (application ID: 2112). Reporting of this project adheres to the Strengthening the Reporting of Observational Studies in Epidemiology (STROBE) and its extension, the REporting of studies Conducted using Observational Routinely-Collected health Data (RECORD) statements.

### Retinal imaging

Macula-centred color fundus photography (CFP) and optical coherence tomography (OCT) imaging were acquired from participants in both AlzEye and UKB using the Topcon 3D OCT-2000 and 3D-OCT 1000 MKII respectively (Topcon Corporation, Tokyo, Japan). Automated image analysis of 45-degree CFPs was performed through the open-source fully-automated deep learning-based model, AutoMorph [26]. AutoMorph takes a raw CFP file, pre-processes the image, segments the optic nerve and retinal vasculature and outputs a table of clinically relevant features. Features regarding optic nerve morphology (optic disc and cup height and width) and retinovascular features corresponding to arterioles and venular morphology were used. Optical coherence tomography images covered a 6.0 mm × 6.0 mm^2^ area and had 128 horizontal B scans with 512 A scans per B scan [27]. We used images from both eyes, where available. In UKB, we included participants who had retinal imaging acquired at the initial assessment visit (baseline instance). Retinal sublayer thicknesses were estimated from OCT using the proprietary software, Topcon Advanced Boundary Segmentation Tool (TABS) [28,29]. Sublayer thicknesses were averaged among the parafoveal regions for inner retinal sublayers: the macular retinal nerve fibre layer (mRNFL), macular ganglion cell - and inner plexiform layer (mGCIPL) and macular inner nuclear layer (mINL). For the macular outer retinal layers of photoreceptor segment layer (mPSL, defined from the INL-outer plexiform interface to the retinal pigment epithelium interdigitation zone) and retinal pigment epithelium-Bruch’s membrane layer (RPE-BM), thicknesses were averaged for the foveal and parafoveal grids. TABS provides additional metadata for each image to establish scan quality based on segmentation error, movement artefact and poor quality. To harmonise paired imaging (both CFP and OCT are taken at the same time on the aforementioned devices), we only included images where the following quality control criteria were met for both CFP and OCT. For CFP, using the AutoMorph in-built image quality grading module, we only included gradable (‘good’ or ‘ok’) images. For OCT, we excluded the poorest 10% of images based on specific image quality control metadata previously described [7,28,30]. The same quality control methods were applied to both cohort datasets.

### Systemic and ocular disease variables

Atrial fibrillation was defined using a combination of self-report on questionnaire (only available in UKB) and hospital admissions data from Hospital Episode Statistics (HES), a national repository of all hospital admissions in England under the provisions of the NHS (at least 97% of hospital admissions in England). Atrial fibrillation was defined as a HES episode with a 10th revision of the International Classification of Diseases (ICD-10) code I48 [24,31] or self-report by the participant of AF specifically. For investigations into prevalent AF, we only included retinal images which postdated a hospital admission with a recording of AF. In UKB, we included images postdating either a hospital admission with AF or the participant self-reporting a history of AF. For defining incident AF in UKB, we excluded those who either self-reported having AF at their initial assessment visit or had a hospital admission with AF coded before their retinal imaging. We then used the first hospital admission with an ICD-10 code indicating AF as the time of disease onset. Major adverse cardiovascular events (MACE), including myocardial infarction, stroke and heart failure were defined using codes I11, I13, I21-I22, I50, I60, I61, I63, I64 [32,33]. Secondary exposure variables included age, sex, ethnicity, hypertension, diabetes mellitus, alcohol consumption (UKB only) and smoking status (UKB only). Ethnicity, as self-reported by participants, was aggregated into four groups as defined by the UK Census [34]. Hypertension and diabetes mellitus were defined using HES diagnostic codes for the AlzEye analysis and through self-report at the initial assessment visit touchscreen questionnaire for UKB.

### Statistical analysis

We analysed data distributions numerically and visually with summary statistics and quantile-quantile plots. Continuous variables were compared between groups using the Mann-Whitney-Wilcoxon test and categorical variables through the U-Statistic permutation test of independence [35]. To examine the association between prevalent AF (primary exposure) and retinal morphology (outcome), we fitted linear mixed effects models with a random intercept at the individual level to account for eyes nested within participants with degrees of freedom estimated using Satterthwaite’s approximation. Models were adjusted for age, sex, ethnicity, diabetes mellitus and hypertension. To mitigate the contribution of additional cardiovascular disease (e.g., high prevalence of heart failure/previous myocardial infarction) on the relationship between retinal morphology and prevalent AF, we refitted linear mixed effects models restricting to those individuals with no previous MACE in AlzEye and UK Biobank. Given the richer confounder data available in UKB, we replicated the analysis within the UKB additionally adjusting for alcohol consumption and smoking status. Sensitivity analyses were additionally conducted excluding those with self-reported eye disease in UKB.

To examine the association between retinal morphology (exposure) and incident AF (time to AF as outcome), we fitted unadjusted and adjusted frailty models with a gamma-distributed random effect on the intercept at the individual level to estimate cause-specific adjusted hazard ratios (HR). The at-risk period was defined from the date of retinal imaging acquisition to hospital admission with an AF diagnostic code with censoring at the earlier of date of death or conclusion of the data refresh date for our UKB application (1st December 2020).

In the AlzEye cohort, there was missing data for ethnicity (n = 8,555, 16.9%), optic nerve indices (n = 10,460 images, 13.5%) and minimally for retinal sublayer thicknesses (all < 1%). In UKB, there was missing data for alcohol (n = 138, 0.3%), smoking (n = 220, 0.6%), ethnicity (n = 256, 0.6%), optic nerve indices (n = 3,867, 9.8%) and minimally for retinal sublayer thicknesses (all < 0.01%). Assuming data was missing at random, for both datasets we therefore performed multiple imputation with chained equations (ten times, five iterations) using predictive mean matching and multinomial logistic regression for continuous and categorical variables respectively with model coefficients pooled using Rubin’s rules [36,37]. Statistical significance was set at p < 0.05 (two-tailed). All analyses were conducted in R version 4.1.0 (R Core Team, 2021. R Foundation for Statistical Computing, Vienna, Austria) and used the mice, survival and lmer packages [38–40].

## Supporting information

S1 Table Association between retinal morphology and both prevalent and incident atrial fibrillation adjusting for age, sex, ethnicity, hypertension, and diabetes mellitus. CI: confidence interval, GC-IPL: ganglion cell-inner plexiform layer, INL: inner nuclear layer, MACE: major adverse cardiovascular event, mRNFL: macular retinal nerve fibre layer, OCT: optical coherence tomography, PRL: photoreceptor layer, RPE-BM: retinal pigment epithelium-Bruch’s membrane layer, SD: standard deviation.
(DOCX)

S2 Table Sensitivity analysis assessing the association between retinal morphology and prevalent atrial fibrillation in UK Biobank excluding those with previous major adverse cardiovascular events. 38,422 controls (previously 39,013) and 473 cases (previously 526). Models were adjusted for age, sex, ethnicity, hypertension, diabetes mellitus, alcohol consumption and smoking status. CI: confidence interval, GC-IPL: ganglion cell-inner plexiform layer, INL: inner nuclear layer, MACE: major adverse cardiovascular event, mRNFL: macular retinal nerve fibre layer, OCT: optical coherence tomography, PRL: photoreceptor layer, RPE-BM: retinal pigment epithelium-Bruch’s membrane layer, SD: standard deviation.
(DOCX)

S3 Table Association between retinal morphology and prevalent and incident atrial fibrillation in UK Biobank excluding those with self-reported eye disease. 34967 controls (previously 39,013) and 425 cases (previously 526) for prevalent analysis. 34267 controls, and 700 incident cases. Models were adjusted for age, sex, ethnicity, hypertension, diabetes mellitus, alcohol consumption and smoking status.
(DOCX)

S1 File The RECORD statement – checklist of items, extended from the STROBE statement, that should be reported in observational studies using routinely collected health data. (DOCX)

S2 File Membership of the UK Biobank Eye and Vision Consortium. (XLSX)

## Abbreviations

AF: atrial fibrillation
ECG: electrocardiogram
OCT: optical coherence tomography
CFP: color fundus photographs
CVD: cardiovascular diseases
UKB: UK Biobank
mRNFL: macular retinal nerve fibre layer
mGCIPL: macular ganglion cell, and inner plexiform layer
mINL: macular inner nuclear layer
mPSL: macular photoreceptor segment layer
RPE-BM: retinal pigment epithelium and Bruch’s membrane
MACE: major adverse cardiovascular events
