# Supplementary material for: Atrial fibrillation and retinal imaging-based oculomics: A cross-sectional and longitudinal analysis of two large cohorts
Source: PLOS Digit Health. 2026 Sep 10;5(9):e0001661. doi: 10.1371/journal.pdig.0001661 (PMC13561364; doi:10.1371/journal.pdig.0001661)
Supplement: Supplementary file 1 [file pdig.0001661.s006.pdf]

Potential Copyright Issues:

a. Figures 1, 2: Please confirm whether you drew the images / clip-art within the figure panels by hand. If you did not draw the images, please provide (a) a link to the source of the images or icons and their license / terms of use; or (b) written permission from the copyright holder to publish the images or icons under our CC-BY 4.0 license. Alternatively, you may replace the images with open source alternatives. See these open source resources you may use to replace images / clip-art:
- https://commons.wikimedia.org
- https://openclipart.org/

Figures have been arranged using the Figma platform, please see the following link for details regarding the use of the icons included in the figures.
https://help.figma.com/hc/en-us/articles/360042296374-Figma-Community-copyright-and-licensing
https://www.figma.com/legal/community-free-resource-license/

b. Figure 1: please (a) provide a direct link to the base layer of the map (i.e., the country or region border shape) and ensure this is also included in the figure legend; and (b) provide a link to the terms of use / license information for the base layer image or shapefile. We cannot publish proprietary or copyrighted maps (e.g. Google Maps, Mapquest) and the terms of use for your map base layer must be compatible with our CC-BY 4.0 license.
https://help.figma.com/hc/en-us/articles/360042296374-Figma-Community-copyright-and-licensing
https://www.figma.com/legal/community-free-resource-license/
Note: if you created the map in a software program like R or ArcGIS, please locate and indicate the source of the basemap shapefile onto which data has been plotted.

If your map was obtained from a copyrighted source please amend the figure so that the base map used is from an openly available source. Alternatively, please provide explicit written permission from the copyright holder granting you the right to publish the material under our CC-BY 4.0 license.

Please note that the following CC BY licenses are compatible with PLOS license: CC BY 4.0, CC BY 2.0 and CC BY 3.0, meanwhile such licenses as CC BY-ND 3.0 and others are not compatible due to additional restrictions.

If you are unsure whether you can use a map or not, please do reach out and we will be able to help you. The following websites are good examples of where you can source open access or public domain maps:
* U.S. Geological Survey (USGS) - All maps are in the public domain. (http://www.usgs.gov)
* PlaniGlobe - All maps are published under a Creative Commons license so please cite "PlaniGlobe, http://www.planiglobe.com, CC BY 2.0" in the image credit after the caption. (http://www.planiglobe.com/?lang=enl)
* Natural Earth - All maps are public domain. (http://www.naturalearthdata.com/about/terms-of-use/)

5. Please ensure that your Ethics Statement is available in its entirety at the beginning of your Methods section, under a subheading 'Ethics Statement'. It must include:

i) The name(s) of the Institutional Review Board(s) or Ethics Committee(s)

ii) The approval number(s), or a statement that approval was granted by the named board(s)

iii) (for human participants or donors) - A statement that formal consent was obtained (must state whether verbal/written) OR the reason consent was not obtained (e.g., anonymity).

vi) (for child participants) - Please update your Ethics Statement with confirmation that written informed consent was obtained from the parent/guardian of each participant under 18 years of age.

We have updated the ethics statement according to the journal's requirements as following: *The AlzEye study has received institutional and ethical review board approval (REC reference: 18/LO/1163), with individual participant consent waived under an approved exemption; access to confidential patient information was additionally authorised under Section 251 of the NHS Act 2006 (CAG reference: 18/CAG/0111). UK Biobank is conducted under the approval of the North-West Research Ethics Committee (ref: 06/MRE08/75), with specific approval obtained for this project (application ID: 2112). Reporting of this study adheres to the Strengthening the Reporting of Observational Studies in Epidemiology (STROBE) statement and its extension, the REporting of studies Conducted using Observational Routinely-Collected health Data (RECORD) statement.*

6. Please provide separate figure files in .tif or .eps format.

For more information about figure files please see our guidelines:

https://journals.plos.org/digitalhealth/s/figures
https://journals.plos.org/digitalhealth/s/figures#loc-file-requirements

7. Please provide an Author Summary. This should appear in your manuscript between the Abstract (if applicable) and the Introduction, and should be 150–200 words long. The aim should be to make your findings accessible to a wide audience that includes both scientists and non-scientists. Sample summaries can be found on our website under Submission Guidelines:

https://journals.plos.org/digitalhealth/s/submission-guidelines#loc-parts-of-a-submission

We thank the editor for this comment. We have added the following author summary statement to the manuscript:

Atrial fibrillation, one of the most common heart conditions causing an irregular heart rhythm, affects millions of people worldwide and significantly increases the risk of stroke and heart failure. Detecting it early, before symptoms arise, remains a major clinical challenge. In this

study, we explored whether the retina could offer clues about a person's cardiac health. Because the retina shares structural and vascular similarities with the brain and heart, changes visible on routine eye scans may reflect broader changes occurring elsewhere in the body.

Using eye imaging data from over 90,000 individuals across two large independent datasets, we found that people with atrial fibrillation show measurable thinning of specific inner retinal layers, the ganglion cell and inner plexiform layer, and the inner nuclear layer, compared to those without atrial fibrillation. Crucially, individuals with a thinner retina at baseline were significantly more likely to develop atrial fibrillation in the future, even before any cardiac diagnosis had been made, underlining the difficulty of establishing the diagnosis.

These findings suggest that standard, non-invasive retinal scans which are widely used both in eye clinics and community settings, could potentially help identify individuals at elevated cardiac risk, potentially enabling earlier intervention and improved outcomes.

8. We have noticed that you have uploaded Supporting Information files, but you have not included a list of legends. Please add a full list of legends for your Supporting Information files after the references list.

If the reviewer comments include a recommendation to cite specific previously published works, please review and evaluate these publications to determine whether they are relevant and should be cited. There is no requirement to cite these works unless the editor has indicated otherwise.

Additional Editor Comments (if provided):

[Note: HTML markup is below. Please do not edit.]

Reviewers' Comments:

5. Review Comments to the Author

Please use the space provided to explain your answers to the questions above. You may also include additional comments for the author, including concerns about dual publication, research ethics, or publication ethics. (Please upload your review as an attachment if it exceeds 20,000 characters)

Reviewer #1: 1. AF was mainly identified by self-report and hospital records. However, AF can be paroxysmal and may be missed. How did the authors assess the possible effect of outcome misclassification on the results?
We thank Reviewer 1 for this question. We agree that the diagnosis of AF is complicated by the often paroxysmal clinical course and absence of symptoms, especially in early disease

stages. Thus, we have investigated two large, independent cohorts, leveraging the deep phenotyping of UK Biobank, as well as AlzEye, a cohort in a highly diverse central London population. As stated in the limitations, validation studies for an ICD-10 code for AF showed a high positive predictive value, but more modest sensitivity, being suggestive that our effect estimates are smaller than the true estimates. The following statement in the limitation paragraph highlights this argument:
"A 2024 manual validation study however showed high PPV for an ICD-10 code for AF (PPV 99.1%) but more modest sensitivity (77.5%)(doi: 10.1016/j.accpm.2024.101398), echoing findings from a 2013 US-based systematic review across 16 studies (median PPV, 89%, sensitivity 79%)(doi: 10.1002/pds.2317). Thus, while we can be confident of our cases, controls may be affected by AF (even more so given the diagnostic challenge in identifying asymptomatic AF) suggesting our effect measures to underestimate the true association"

2. In AlzEye, several retinal markers were significant, but in UK Biobank the most consistent finding was mainly mGCIPL thinning. How do the authors explain this difference between the two cohorts?
We thank Reviewer 1 for this comment. Thinner mGCIPL was found in both Alzeye and UK Biobank in individuals with prevalent AF as discussed in the second paragraph of the discussion. In UK Biobank we also investigated retinal sublayer thickness in participants with incident AF.

We hypothesise that the pathophysiologic mechanism behind the mGCIPL is transsynaptic retrograde degeneration, as seen in patients with ischemic strokes. Patients with AF have an increased risk of silent cerebral infraction, it is therefore conceivable that the mGCIPL thinning is a consequence of paroxysmal AF with no clinical symptom, thus, highlighting the difficulty of establishing the diagnosis of AF.

To address this comment and a comparable comment by Reviewer 2, we have added the following statement, supported by Khawaja et al (DOI: 10.1016/j.ophtha.2019.08.015)
"The particular sensitivity of the mGCIPL to systemic pathophysiological processes is further supported by an analysis of over 42,000 UK Biobank participants that GCIPL explains approximately double the variance attributable to systemic factors, thus being the superior inner retinal biomarker. The combination of retrograde transsynaptic neurodegeneration from AF-associated cerebrovascular injury and direct ischaemic susceptibility of the high-metabolic-demand ganglion cell layer provides a dual mechanistic basis for the mGCIPL as a hypothetically plausible and reproducible retinal correlate of AF."

3. The models were adjusted for age, sex, ethnicity, hypertension, and diabetes, and in UK Biobank also for smoking and alcohol use. What was the reason for choosing these variables? Were other important AF-related factors, such as BMI, kidney function, blood lipids, medication use, heart failure, or sleep apnea, considered?
Thank you for this great comment. We agree that there are many more variables that one could adjust for. We chose the variables age, sex, ethnicity, hypertension and diabetes for AlzEye and additionally smoking and alcohol use in UK Biobank, as there is a good understanding of the relationship between these biomarkers to retinal morphology, especially

in the context of GCIPL, as shown by Khawaja et al (DOI: 10.1016/j.ophtha.2019.08.015). Furthermore, we would argue that adjusting for additional variables is limited by the predictive power of such variables and the risk of overadjustment bias. (doi: 10.1097/EDE.0b013e3181a819a1). As with other observational health data, we acknowledge that there is a risk of residual confounding. We have also added this into the Limitations.

4. How did the authors rule out the effect of pre-existing eye diseases that might influence OCT or retinal imaging measurements?
Thank you for this comment. We acknowledge the Reviewer's comment and have therefore performed an additional sensitivity analysis excluding individuals with self-reported eye disease in the UK Biobank. Despite the reduced statistical power, our findings were consistent with the primary analyses. We have added these to the manuscript.

Changes made: *Similarly, excluding those with self-reported eye diseases in UKB did not alter the direction or magnitude of any significant effect estimates (Supplementary Table 3).*

Supplementary Table 3 added

5. If both eyes from one patient were included, or if one patient had repeated records, did the authors make sure that the analysis was separated at the patient level? Please clarify how within-patient correlation was handled.
We thank Reviewer 1 for this question. As stated in the Methods section under the heading "Statistical analysis", within-patient correlation was explicitly accounted for by fitting linear mixed effects models with a random intercept at the individual level, which treats the two eyes from each participant as nested observations rather than independent data points. This approach acknowledges that measurements from the same individual are inherently more correlated with one another than with measurements from different individuals, and partitions this variance accordingly before estimating the effect of atrial fibrillation on retinal morphology. Consequently, all inference is made at the patient level, ensuring that the inclusion of bilateral eye data does not artificially inflate the effective sample size or bias the results.

6. Why was mGCIPL the most consistent signal? Please discuss more fully in the Discussion section why mGCIPL may be related to AF.
Thank you for this comment. The mGCIPL's consistency across both cohorts likely reflects the convergence of two distinct mechanistic pathways. The first pathway as discussed is most likely retrograde transsynaptic neurodegeneration, as AF increases the risk of silent cardioembolic infarctions affecting the posterior visual pathway. This mirrors mGCIPL thinning established after ischaemic stroke and in multiple sclerosis. Secondly, Khawaja et al. (2020) (DOI: 10.1016/j.ophtha.2019.08.015) demonstrated that GCIPL explains 11.2% of variance attributable to systemic factors and showed the most significant associations with IOP and ageing. The authors concluded that GCIPL is the superior inner retinal biomarker for systemic pathophysiological processes. This inherent sensitivity likely explains why mGCIPL, rather than other retinal parameters, reproduced across two cohorts with markedly

different characteristics. We have added the following statement at the end of the paragraph discussing mGCIPL thinning:

"The particular sensitivity of the mGCIPL to systemic pathophysiological processes is further supported by an analysis of over 42,000 UK Biobank participants that GCIPL explains approximately double the variance attributable to systemic factors, thus being the superior inner retinal biomarker. To contextualise these effect sizes, the -1.96 µm mGCIPL thinning observed in the AlzEye cohort represents 37.8% of the mGCIPL standard deviation and is equivalent to approximately 13 years of age-related thinning, and approximately 4 times the mGCIPL thinning associated with daily alcohol consumption. The -1.19 µm difference in the UKB cohort similarly represents 22.9% of the standard deviation and is equivalent to approximately 8 years of aging. The combination of retrograde transsynaptic neurodegeneration from AF-associated cerebrovascular injury and direct ischaemic susceptibility of the high-metabolic-demand ganglion cell layer provides a dual mechanistic basis for the mGCIPL as a hypothetically plausible and reproducible retinal correlate of AF.The combination of retrograde transsynaptic neurodegeneration from AF-associated cerebrovascular injury and direct ischaemic susceptibility of the high-metabolic-demand ganglion cell layer provides a dual mechanistic basis for the mGCIPL as a hypothetically plausible and reproducible retinal correlate of AF."

7. ECG, wearable devices, and pulse screening (PPG) are already available for AF detection. Compared with these existing methods, what is the added value of retinal imaging for AF screening?

We thank Reviewer 1 for this comment. We agree that ECG, wearables, and photoplethysmography (PPG) are established and evolving tools for AF screening. However, we respectfully submit that retinal imaging occupies a fundamentally different and complementary niche rather than competing with these modalities.

Rhythm diagnostics based on ECG, continuous ambulatory monitors, or smartphone PPG detect AF only when the arrhythmia is electrically active at the time of recording. This represents a critical limitation for paroxysmal or subclinical AF, which may be entirely absent during monitoring windows. The 2024 ESC Guidelines state that population-based ECG screening for AF yields no benefit at a single timepoint, and even implantable loop recorder-detected AF in the LOOP trial did not translate to a reduction in stroke. Moreover, the ESC Guidelines explicitly classify PPG as "non-diagnostic" as ECG physician confirmation is mandated before AF management is initiated (Van Gelder et al. 2024).

In contrast, retinal imaging detects cumulative structural consequences of AF with microvascular and neurodegenerative end-organ damage are present regardless of whether the arrhythmia is occurring at the moment of imaging. As we state in the Discussion, retinal findings are proposed as a potential complementary tool that could guide clinicians toward more intensive cardiac investigation such as implantable loop recorders in clinically equivocal cases where intermittent rhythm monitoring would otherwise be unrevealing.

Furthermore, retinal imaging infrastructure is already embedded in primary care optometry across developed nations, offering a scalable, opportunistic, non-invasive route to AF-related end-organ assessment without requiring patient cooperation with prolonged rhythm monitoring devices. These findings of course need to be confirmed in subsequent studies.

We have added the following statement to the discussion:

"While the observed retinovascular alterations and retinal sublayer thickness changes are consistent with our pathomechanistic hypothesis of a causal link to AF, their clinical context

is paramount. The diagnosis of AF currently relies on cardiac monitoring, clinical presentation, and cardiovascular risk profiling. The limitations of ECG monitoring over a prolonged period is complicated by the fact that photoplethysmography-based monitoring is constrained by evidence quality and the requirement for physician ECG confirmation before diagnosis (Van Gelder et al.2024). Retinal imaging in comparison, captures structural end-organ sequelae of AF that persist independently of arrhythmia burden at any single timepoint. We propose that these retinal findings are not a substitute for this paradigm, but rather a complementary tool. From an oculomic perspective, they highlight the complex systemic nature of AF and, if confirmed in subsequent studies assessing their predictive potential, could guide clinicians to pursue more extensive cardiac investigations, such as implantable loop recorders, in clinically equivocal cases.

Reviewer #2: This manuscript investigates the association between multimodal retinal imaging parameters and both prevalent and incident atrial fibrillation (AF) using data from two large cohorts (AlzEye and UK Biobank). The study leverages quantitative retinal phenotyping derived from optical coherence tomography and fundus photography. The central finding is an association between thinner macular ganglion cell-inner plexiform layer (mGCIPL) and AF, which was replicated across cohorts.
Overall, this is a strong and timely study at the intersection of ophthalmic imaging and cardiovascular disease. The use of large, well-characterised cohorts and advanced retinal phenotyping is a key strength, and replication across datasets supports the robustness of the findings. However, to strengthen the paper, I recommend some revisions as outlined below. Addressing these issues will improve both the clarity and overall quality of the study.
Many thanks for this feedback.

Major comments:
1. The manuscript would benefit from a more cautious framing of its mechanistic and clinical implications. Several statements appear to extend beyond what can be directly supported by the presented observational data.
We thank Reviewer 2 for this comment. We have therefore rephrased the mentioned statements as below:

For example,
• "our report… highlights the mechanistic and potential prognostic utility of retinal imaging in cardiac arrhythmias" (discussion, first paragraph) is an overstatement in the absence of any validation of clinical performance.
"Our report, which represents to our knowledge by some magnitude the largest reported analysis of retinal morphology in people with AF, underlines the mechanistic associations of AF and structural retinal changes and, once validated by further studies could support potential prognostic utility of retinal imaging in cardiac arrhythmias."

• "observed… changes are consistent with our pathomechanistic hypothesis of a causal link to AF" (discussion, fourth paragraph) risk implying a directional causal interpretation that cannot be established from cross-sectional and observational associations alone.

“*While the observed retinovascular alterations and retinal sublayer thickness changes are consistent with our pathomechanistic hypothesis of a clinical association with AF, their clinical context is paramount.”*

• “these retinal findings are not a substitute for this paradigm, but rather a complementary tool” (discussion, fourth paragraph) overstate translational readiness given that diagnostic accuracy, incremental predictive value, and clinical utility have not been evaluated.
“We propose that these retinal findings are not a substitute for this paradigm, but rather a complementary tool. From an oculomic perspective, they highlight the complex systemic nature of AF and, if confirmed in subsequent studies assessing their predictive potential, could guide clinicians to pursue more extensive cardiac investigations, such as implantable loop recorders, in clinically equivocal cases.”

• Implying “early detection,” “risk stratification,” or the ability of retinal imaging to “directly assess end-organ damage” and provide insight into AF pathophysiology also appear premature. (discussion, final paragraph) This is beyond the scope of the current analysis.
Thank you for this comment. We have rephrased the last paragraph as following:
“*This study demonstrates that individuals with prevalent and incident AF exhibit thinner mGCIPL and mINL, alongside differences in vascular indices. The observed mGCIPL thinning may result from microvascular events associated with AF that affect the visual pathways and retina. In contrast, the novel finding of mINL thinning* ***could be interpreted in the context of*** *underlying subclinical ischemia reflecting systemic cardiovascular compromise. These findings,* ***once validated in subsequent studies****, highlight the potential for non-invasive retinal imaging to provide early detection and risk stratification of AF, as well as an easily-accessible opportunity to understand AF pathophysiology and directly assess end-organ damage. Given the widespread availability of retinal imaging in primary care optometrist stores high streets across developed nations, further attention is warranted to explore the role of retina imaging for earlier diagnosis of AF, and the opportunity to target therapy addressing underlying vascular disease to reduce the large patient and healthcare burdens associated with AF.”*

• “the novel finding of mINL thinning suggests underlying subclinical ischemia reflecting systemic cardiovascular compromise” introduces a mechanistic interpretation that exceeds what can be directly inferred from the observed association. A more neutral description of mINL thinning as an associated imaging finding, without attributing it to subclinical ischemia or systemic cardiovascular compromise, would better align with the study design.
The authors are encouraged to align the discussion more closely with the observed associations and to avoid extending the interpretation into diagnostic, prognostic, or mechanistic claims that are not directly demonstrated.

We thank Reviewer 2 for this comment. We fully agree that the novel finding of thinning of the mINL should be interpreted carefully in the context of AF, thus we have discussed this in the third paragraph in more detail.

Firstly, transsynaptic retrograde degeneration does not affect the mINL, this is supported by Balk et al (DOI: 10.1136/jnnp-2014-308189) and a systematic review and meta-analysis by

Chan et al (DOI: 10.1016/j.ophtha.2018.08.009). Furthermore, the mINL receives its blood supply from the retinal deep capillary plexus and the intermediate plexus, and given the highly metabolic environment in the macula area, is very susceptible to subclinical ischaemia. This may present in retinal ischaemic presentations such as paracentral acute middle maculopathy, a disease highly associated with cardiovascular risk burden (DOI:10.1016/j.preteyeres.2020.100884, DOI: 10.1016/j.ajo.2024.08.005) but also in retinal ischaemic perivascular lesions (DOI: 10.1007/s40123-025-01148-5). These retinal sublayer manifestations associated with cardiovascular disease burden causing a thinning of mINL have been shown to be more prevalent in AF (doi: 10.1161/JAHA.122.028853).

In the context of cardiovascular disease and atrial fibrillation, which is considered a thrombo-inflammatory condition (DOI:10.1016/S0140-6736(09)60040-4), we believe that this interpretation is a viable hypothesis that needs to be confirmed in subsequent studies, although we appreciate that this may be difficult to achieve.

By showing the finding of mINL in both large scale cohorts with different risk profiles, we feel that this interpretation is justified, we however agree that the wording could be improved. We have therefore adjusted the final paragraph of the discussion as following:

“In contrast, the novel finding of mINL thinning could be interpreted in the context of underlying subclinical ischemia reflecting systemic cardiovascular compromise. These findings, once validated in subsequent studies, highlight the potential for non-invasive retinal imaging to provide early detection and risk stratification of AF, as well as an easily-accessible opportunity to understand AF pathophysiology and directly assess end-organ damage.”

2. The adjustment strategy differs between cohorts, with additional lifestyle covariates (smoking and alcohol consumption) included in the UK Biobank models but not in AlzEye. Could the authors clarify the rationale for this discrepancy? If this reflects differences in data availability, it would be helpful to state this explicitly in the Methods section. The authors may want to consider presenting both approaches of adjustment (with and without these additional adjustments in UK Biobank) to aid comparability.
We thank Reviewer 2 for this observation. The reason for the additional analysis in UKB is based on data availability in AlzEye, we have clarified this in the Methods section as following:
“*Given the richer confounder data available in UKB, we replicated the analysis within the UKB additionally adjusting for alcohol consumption and smoking status.”*

In regards to the Reviewer’s point, about providing consistent adjustment approaches across both cohorts for comparability, we have now done this with an additional table (Supplementary Table 1) and amendment to the Results section:

Replicating the analysis in the UKB cohort of 526 individuals with prevalent AF using the same variable adjustment (Supplementary Table 1) and additionally adjusting for alcohol consumption and smoking status, effect estimates were of the same direction as AlzEye, with smaller magnitudes of difference (Table 3)

Supplementary Table 1 added

In addition, consideration could be given to other potential confounders such as body mass index, socioeconomic status, medication use, treatment status, and duration of atrial fibrillation, if available. If such variables cannot be incorporated, the potential for residual confounding should be more explicitly acknowledged and discussed as a limitation.

We thank Reviewer 2 for the comment, and would like to refer to the response to Reviewer 1, which is copied below:

Thank you for this great comment. We agree that there are many more variables that one could adjust for. We chose the variables age, sex, ethnicity, hypertension and diabetes for AlzEye and additionally smoking and alcohol use in UK Biobank, as there is a good understanding of the relationship between these biomarkers to retinal morphology, especially in the context of GCIPL, as shown by Khawaja et al (DOI: 10.1016/j.ophtha.2019.08.015). Furthermore, we would argue that adjusting for additional variables is limited by the predictive power of such variables and the risk of overadjustment bias. (doi: 10.1097/EDE.0b013e3181a819a1). As with other observational health data, we acknowledge that there is a risk of residual confounding. We have also added this into the LImitations.

3. The magnitude and clinical significance of the reported associations require clearer contextualisation in the discussion section. While statistically significant, the observed differences in retinal layer thickness (e.g., -1.96 µm in mGCIPL [AlzEye cohort] and -1.19 µm in mGCIPL [UK Biobank cohort]) appear small, and their relevance relative to measurement variability/ inherent noise, age-related changes, and clinical decision-making thresholds is not fully addressed. A more explicit discussion of effect size interpretation would strengthen the translational framing of the work.
We thank Reviewer 2 for this important comment. Based on previously referenced work by Khawaja et al (DOI: 10.1016/j.ophtha.2019.08.015), we have now added the following statement to put into context and to support the translational relevance of our findings.
“To contextualise these effect sizes, the -1.96 µm mGCIPL thinning observed in the AlzEye cohort represents 37.8% of the mGCIPL standard deviation and is equivalent to approximately 13 years of age-related thinning, and approximately 4 times the mGCIPL thinning associated with daily alcohol consumption. The -1.19 µm difference in the UKB cohort similarly represents 22.9% of the standard deviation and is equivalent to approximately 8 years of aging.”

4. In the UK Biobank incidence analyses, the relatively short interval between baseline imaging and AF ascertainment may raise the possibility that a proportion of cases had undiagnosed or subclinical AF at baseline. The authors may wish to acknowledge this as a potential limitation. Additionally, the authors could consider performing sensitivity analyses excluding early incident cases (if date of first recorded diagnosis is available), which may help to further clarify the temporal interpretation of the findings.

We thank the Reviewer for this point. Unfortunately, excluding those within incident AF within 24 months reduces the sample size considerably with wide resulting intervals. We have accordingly amended the Discussion to explicitly mention this as a limitation.

In the incident AF analysis, approximately 20% of individuals developed AF within 24 months of retinal imaging. Given that AF is typically paroxysmal in its early stages, our findings may be more applicable to those with prevalent rather than incident AF.

Minor comments:
1. In the UK Biobank analyses, sensitivity analyses restricting to individuals without prior major adverse cardiovascular events (MACE), as performed in the AlzEye cohort, were not presented. This may reflect the relatively small number of AF cases within UK Biobank after such restriction, limiting statistical power. It would be helpful for the authors to explicitly acknowledge this in the Methods/Results section to clarify why this analysis was not replicated across cohorts.

Thank you for this comment. Our main rationale for this was that the prevalence of previous major adverse cardiovascular events in the UK Biobank cohort was low. However, we acknowledge the Reviewer's point and have therefore additionally conducted this analysis and added a Supplementary Table and amended the Results section. This showed findings consistent with the primary analysis.

Changes made: *Sensitivity analysis excluding those with previous MACE in UK Biobank did not result in any significant change in effect estimates. Individuals with prevalent AF had significantly thinner mGCIPL (-1.15 microns, 95% CI: -1.72, -0.58, n prevalent AF = 473, Supplementary Table 2).*

Supplementary Table 2 added.

Reviewer #3: Congratulations to the authors, very interesting work. This report is well structured investigating the association between retinal imaging parameters with prevalent and incident AF. Specific minor comments are provided below:
We thank Reviewer 3 for their kind comment.

Minor comments:
1. Caption figure 3: Please be consistent with the abbreviation of 'AF'
Corrected

2. Table 1 and 2: Please check missing units in metrics and be consistent 'n', 'N'
Corrected
3. Caption Table 3: Please can double check typo at the end of the caption: '3'

4. Discussion: The term 'high street' imaging is used; perhaps use a different terminology.
We thank Reviewer 3 for this comment, we have changed the term high street to primary care optometrist stores.

5. Methods: Clarify if the retinal measurements were taken from a single eye (and if so, how it was selected) or an average of both eyes.
We thank Reviewer 3 for this question, and would like to refer to the answer provided to Reviewer 1 and copied below:

As stated in the Methods section under the heading “Statistical analysis”, within-patient correlation was explicitly accounted for by fitting linear mixed effects models with a random intercept at the individual level, which treats the two eyes from each participant as nested observations rather than independent data points. This approach acknowledges that measurements from the same individual are inherently more correlated with one another than with measurements from different individuals, and partitions this variance accordingly before estimating the effect of atrial fibrillation on retinal morphology. Consequently, all inference is made at the patient level, ensuring that the inclusion of bilateral eye data does not artificially inflate the effective sample size or bias the results.
