# Supplementary material for: Atrial fibrillation and retinal imaging-based oculomics: A cross-sectional and longitudinal analysis of two large cohorts
Source: PLOS Digit Health. 2026 Sep 10;5(9):e0001661. doi: 10.1371/journal.pdig.0001661 (PMC13561364; doi:10.1371/journal.pdig.0001661)
Supplement: Supplementary file 2 [file pdig.0001661.s007.pdf]

Review Comments to the Author

Reviewer #1: 1. AF was mainly identified by self-report and hospital records. However, paroxysmal AF and asymptomatic AF can easily be missed. Therefore, some participants in the control group may have had undiagnosed AF. This could affect the comparison between AF and non-AF groups. The authors should further discuss in the Discussion section how this outcome misclassification may affect the direction and size of the effect estimates.

We thank the reviewer for highlighting this point. We therefore have added the following statement to the discussion:

*“**Because paroxysmal and asymptomatic AF will likely not be included in hospital coding or self-reports, some individuals classified as controls, and in the incident analysis, may in fact have had undiagnosed AF at the time of retinal imaging.** Thus, while we can be confident of our cases, controls may be affected by AF (even more so given the diagnostic challenge in identifying asymptomatic AF) suggesting our effect measures to underestimate the true association.“*

2. The models adjusted for age, sex, ethnicity, hypertension, and diabetes, and the UK Biobank models also adjusted for smoking and alcohol use. However, both AF and retinal structure may also be affected by BMI, blood lipids, kidney function, heart failure, sleep apnea, medication use, and socioeconomic status. The authors should clearly state whether these variables were available. If they could not be included, the authors should discuss residual confounding more fully as a limitation.

We thank the reviewer for this comment. We agree that there are upstream variables (e.g. sleep apnea) which may be a source of residual confounding, as with most observational health data analyses. We have added the following statement to the limitations.

"Finally, as with other observational health data analyses, there may be factors which we could not account for but may plausibly influence both AF risk and retinal structure (residual confounding)."

Reviewer #2: I thank the authors for their comprehensive and thoughtful revision. The manuscript has improved considerably. In particular, the addition of multiple sensitivity analyses (i.e., excluding eye disease and MACE), clarification of the statistical modelling, better contextualisation of effect sizes, and a more nuanced discussion have substantially strengthened the paper.

I only have two minor comments.

1. I appreciate that the authors now acknowledge that approximately 20% of participants developed AF within 24 months of retinal imaging and because AF is typically paroxysmal in its early stages, the findings may be more applicable to prevalent than incident AF. Given this important caveat, the authors may consider softening the concluding statements. One example: the statement that “This study demonstrates that individuals with prevalent and incident AF exhibit thinner mGCIPL and mINL, alongside differences in vascular indices” appears somewhat stronger than the preceding discussion. A wording that better reflects the uncertainty surrounding the incident analyses may improve consistency.

We thank the reviewer for this suggestion and have adapted the concluding sentence to better reflect the asymmetry between the prevalent and incident findings.

"This study demonstrates that individuals with prevalent AF exhibit thinner mGCIPL and mINL, alongside differences in vascular indices, with a similar but less consistent pattern observed for incident AF."

2. I appreciate that the limitations section now acknowledges the possibility of residual confounding. The authors may wish to expand this slightly by noting that factors such as medication use (particularly anticoagulants), body mass index, chronic kidney disease or kidney function, and AF duration could plausibly influence both retinal morphology and AF status. Explicitly acknowledging these potential sources of residual confounding would further strengthen the discussion.

Many thanks for this comment. We have addressed this now in the limitations adding the following statement:

"Finally, as with other observational health data analyses, there may be factors which we could not account for but may plausibly influence both AF risk and retinal structure (residual confounding)."

Reviewer #3: Congratulations to the authors, overall clarity of the work has been improved in this updated version.

Many thanks for your kind comment.
