# Supplementary material for: Atrial fibrillation and retinal imaging-based oculomics: A cross-sectional and longitudinal analysis of two large cohorts
Source: PLOS Digit Health. 2026 Sep 10;5(9):e0001661. doi: 10.1371/journal.pdig.0001661 (PMC13561364; doi:10.1371/journal.pdig.0001661)
Supplement: Supplementary file 3 [file pdig.0001661.s001.docx]

|  | | **Prevalent Atrial Fibrillation**  **(n prevalent AF = 526)** | | **Incident Atrial Fibrillation**  **(n incident AF= 838)** | |
| --- | --- | --- | --- | --- | --- |
| **Characteristic** | | **Estimate (95% CI)** | ***p-*value** | **HR (95% CI)** | ***p*-value** |
| **Retinovascular**  **(per SD)** | Arteriolar calibre | 0.03 (-0.05, 0.11) | 0.46 | 0.96 (0.83, 1.10) | 0.54 |
|  | Arteriolar distance tortuosity | -0.05 (-0.12, 0.02) | 0.20 | 1.00 (0.92, 1.09) | 0.98 |
|  | Arteriolar fractal dimension | 0.07 (-0.01, 0.14) | 0.09 | 1.00 (0.88, 1.13) | 0.94 |
|  | Venular calibre | -0.03 (-0.11, 0.05) | 0.51 | 0.90 (0.79, 1.04) | 0.15 |
|  | Venular distance tortuosity | -0.07 (-0.14, 0.01) | 0.07 | 0.96 (0.88, 1.05) | 0.40 |
|  | Venular fractal dimension | -0.03 (-0.10, 0.05) | 0.52 | 1.01 (0.89, 1.15) | 0.84 |
| **Optic nerve morphology**  **(per SD)** | Cup height | 0.06 (-0.02, 0.14) | 0.17 | 0.98 (0.86, 1.13) | 0.82 |
|  | Cup width | 0.08 (0.00, 0.17) | 0.05 | 0.98 (0.86, 1.12) | 0.76 |
|  | Disc height | 0.06 (-0.02, 0.14) | 0.13 | 0.99 (0.87, 1.12) | 0.82 |
|  | Disc width | 0.06 (-0.02, 0.15) | 0.12 | 0.97 (0.86, 1.09) | 0.59 |
| **OCT**  **(μm)** | mRNFL | -0.18 (-0.48, 0.12) | 0.25 | 1.08 (0.92, 1.27) | 0.36 |
|  | mGCIPL | -1.19 (-1.73, -0.65) | **1.5 x 10^-5^** | 1.30 (1.06, 1.56) | **0.009** |
|  | mINL | -0.18 (-0.40, 0.05) | 0.12 | 0.98 (0.81, 1.19) | 0.86 |
|  | PRL | -0.23 (-1.00, 0.54) | 0.56 | 0.99 (0.79, 1.25) | 0.95 |
|  | RPE-BM | 0.21 (-0.11, 0.52) | 0.19 | 1.02 (0.81, 1.28) | 0.86 |

Supplementary Table 1: Association between retinal morphology and both prevalent and incident atrial fibrillation adjusting for age, sex, ethnicity, hypertension, and diabetes mellitus. CI: confidence interval, GC-IPL: ganglion cell-inner plexiform layer, INL: inner nuclear layer, MACE: major adverse cardiovascular event, mRNFL: macular retinal nerve fibre layer, OCT: optical coherence tomography, PRL: photoreceptor layer, RPE-BM: retinal pigment epithelium-Bruch’s membrane layer, SD: standard deviation.
