# Supplementary material for: Atrial fibrillation and retinal imaging-based oculomics: A cross-sectional and longitudinal analysis of two large cohorts
Source: PLOS Digit Health. 2026 Sep 10;5(9):e0001661. doi: 10.1371/journal.pdig.0001661 (PMC13561364; doi:10.1371/journal.pdig.0001661)
Supplement: Supplementary file 4 [file pdig.0001661.s002.docx]

|  | | **Prevalent Atrial Fibrillation**  **(n prevalent AF = 473)** | |
| --- | --- | --- | --- |
| **Characteristic** | | **Estimate (95% CI)** | ***p-*value** |
| **Retinovascular**  **(per SD)** | Arteriolar calibre | 0.02 (-0.07, 0.10) | 0.73 |
|  | Arteriolar distance tortuosity | -0.05 (-0.13, 0.02) | 0.18 |
|  | Arteriolar fractal dimension | 0.07 (-0.02, 0.15) | 0.11 |
|  | Venular calibre | -0.03 (-0.12, 0.05) | 0.44 |
|  | Venular distance tortuosity | -0.06 (-0.14, 0.02) | 0.11 |
|  | Venular fractal dimension | -0.03 (-0.11, 0.05) | 0.45 |
| **Optic nerve morphology**  **(per SD)** | Cup height | 0.04 (-0.04, 0.13) | 0.34 |
|  | Cup width | 0.07 (-0.02, 0.15) | 0.13 |
|  | Disc height | 0.05 (-0.04, 0.13) | 0.27 |
|  | Disc width | 0.05 (-0.03, 0.14) | 0.22 |
| **OCT**  **(μm)** | mRNFL | -0.26 (-0.57, 0.06) | 0.11 |
|  | mGCIPL | -1.15 (-1.72, -0.58) | **7.4 x 10^-5^** |
|  | mINL | -0.15 (-0.39, 0.09) | 0.21 |
|  | PRL | -0.12 (-0.93, 0.69) | 0.77 |
|  | RPE-BM | 0.24 (-0.09, 0.57) | 0.16 |

Supplementary Table 2: Sensitivity analysis assessing the association between retinal morphology and prevalent atrial fibrillation in UK Biobank excluding those with previous major adverse cardiovascular events. . 38,422 controls (previously 39,013) and 473 cases (previously 526). Models were adjusted for age, sex, ethnicity, hypertension, diabetes mellitus, alcohol consumption and smoking status. CI: confidence interval, GC-IPL: ganglion cell-inner plexiform layer, INL: inner nuclear layer, MACE: major adverse cardiovascular event, mRNFL: macular retinal nerve fibre layer, OCT: optical coherence tomography, PRL: photoreceptor layer, RPE-BM: retinal pigment epithelium-Bruch’s membrane layer, SD: standard deviation.
