# Supplementary material for: Atrial fibrillation and retinal imaging-based oculomics: A cross-sectional and longitudinal analysis of two large cohorts
Source: PLOS Digit Health. 2026 Sep 10;5(9):e0001661. doi: 10.1371/journal.pdig.0001661 (PMC13561364; doi:10.1371/journal.pdig.0001661)
Supplement: Supplementary file 5 [file pdig.0001661.s003.docx]

|  | | **Prevalent Atrial Fibrillation**  **(n prevalent AF = 425)** | | **Incident Atrial Fibrillation**  **(n incident AF= 700)** | |
| --- | --- | --- | --- | --- | --- |
| **Characteristic** | | **Estimate (95% CI)** | ***p-*value** | **HR (95% CI)** | ***p*-value** |
| **Retinovascular**  **(per SD)** | Arteriolar calibre | 0.02 (-0.07, 0.10) | 0.73 | 0.94 (0.80, 1.09) | 0.40 |
|  | Arteriolar distance tortuosity | -0.05 (-0.13, 0.02) | 0.18 | 1.01 (0.92, 1.11) | 0.81 |
|  | Arteriolar fractal dimension | 0.07 (-0.02, 0.15) | 0.11 | 0.97 (0.85, 1.11) | 0.67 |
|  | Venular calibre | -0.03 (-0.12, 0.05) | 0.44 | 0.90 (0.78, 1.04) | 0.16 |
|  | Venular distance tortuosity | -0.06 (-0.14, 0.02) | 0.11 | 0.97 (0.88, 1.06) | 0.49 |
|  | Venular fractal dimension | -0.03 (-0.11, 0.05) | 0.45 | 1.02 (0.89, 1.17) | 0.79 |
| **Optic nerve morphology**  **(per SD)** | Cup height | 0.04 (-0.04, 0.13) | 0.34 | 0.97 (0.84, 1.12) | 0.70 |
|  | Cup width | 0.07 (-0.02, 0.15) | 0.13 | 0.96 (0.83, 1.10) | 0.54 |
|  | Disc height | 0.05 (-0.04, 0.13) | 0.27 | 0.97 (0.86, 1.11) | 0.68 |
|  | Disc width | 0.05 (-0.03, 0.14) | 0.22 | 0.96 (0.85, 1.09) | 0.52 |
| **OCT**  **(μm,**  **Per SD increase for prevalent**  **Per SD decrease for incident)** | mRNFL | -0.26 (-0.57, 0.06) | 0.11 | 1.08 (0.78, 1.31) | 0.46 |
|  | mGCIPL | -1.15 (-1.72, -0.58) | **7.4 x 10^-5^** | 1.34 (1.07, 1.67) | **0.009** |
|  | mINL | -0.15 (-0.39, 0.09) | 0.21 | 1.05 (0.84, 1.31) | 0.69 |
|  | PRL | -0.12 (-0.93, 0.64) | 0.77 | 1.03 (0.80, 1.32) | 0.81 |
|  | RPE-BM | 0.24 (-0.09, 0.57) | 0.16 | 1.02 (0.75, 1.38) | 0.90 |

Supplementary Table 3: Association between retinal morphology and prevalent and incident atrial fibrillation in UK Biobank excluding those with self-reported eye disease. 34967 controls (previously 39,013) and 425 cases (previously 526) for prevalent analysis. 34267 controls, and 700 incident cases. Models were adjusted for age, sex, ethnicity, hypertension, diabetes mellitus, alcohol consumption and smoking status.
