# Supplementary material for: Atrial fibrillation and retinal imaging-based oculomics: A cross-sectional and longitudinal analysis of two large cohorts
Source: PLOS Digit Health. 2026 Sep 10;5(9):e0001661. doi: 10.1371/journal.pdig.0001661 (PMC13561364; doi:10.1371/journal.pdig.0001661)
Supplement: Peer review history

# Peer review: Decision Letter 0

**DOI:** 10.1371/journal.pdig.0001661.r001

**Authors:** Feng Liu, Kaisaierjiang Kadier

16 Apr 2026

Response to Reviewers Revised Manuscript with Track Changes Manuscript

**Journal Requirements:**

1. Please amend your detailed Financial Disclosure statement. This is published with the article. It must therefore be completed in full sentences and contain the exact wording you wish to be published.

i. Please clarify all sources of financial support for your study. List the grants, grant numbers, and organizations that funded your study, including funding received from your institution. Please note that suppliers of material support, including research materials, should be recognized in the Acknowledgements section rather than in the Financial Disclosure.

ii. State the initials, alongside each funding source, of each author to receive each grant. For example: "This work was supported by the National Institutes of Health (####### to AM; ###### to CJ) and the National Science Foundation (###### to AM)."

iii. State what role the funders took in the study. If the funders had no role in your study, please state: “The funders had no role in study design, data collection and analysis, decision to publish, or preparation of the manuscript.”

iv. If any authors received a salary from any of your funders, please state which authors and which funders.

2. Please send a completed 'Competing Interests' statement, including any COIs declared by your co-authors. If you have no competing interests to declare, please state "The authors have declared that no competing interests exist". Otherwise please declare all competing interests beginning with the statement "I have read the journal's policy and the authors of this manuscript have the following competing interests:"

3. In this instance it seems there may be acceptable restrictions in place that prevent the public sharing of your minimal data. However, in line with our goal of ensuring long-term data availability to all interested researchers, PLOS’ Data Policy states that authors cannot be the sole named individuals responsible for ensuring data access (http://journals.plos.org/plosone/s/data-availability#loc-acceptable-data-sharing-methods).

Data requests to a non-author institutional point of contact, such as a data access or ethics committee, helps guarantee long term stability and availability of data. Providing interested researchers with a durable point of contact ensures data will be accessible even if an author changes email addresses, institutions, or becomes unavailable to answer requests.

Before we proceed with your manuscript, please also provide non-author contact information (phone/email/hyperlink) for a data access committee, ethics committee, or other institutional body to which data requests may be sent. If no institutional body is available to respond to requests for your minimal data, please consider if there any institutional representatives who did not collaborate in the study, and are not listed as authors on the manuscript, who would be able to hold the data and respond to external requests for data access? If so, please provide their contact information (i.e., email address). Please also provide details on how you will ensure persistent or long-term data storage and availability.

4. Some material included in your submission may be copyrighted. According to PLOS’s copyright policy, authors who use figures or other material (e.g., graphics, clipart, maps) from another author or copyright holder must demonstrate or obtain permission to publish this material under the Creative Commons Attribution 4.0 International (CC BY 4.0) License used by PLOS journals. Please closely review the details of PLOS’s copyright requirements here: PLOS Licenses and Copyright. If you need to request permissions from a copyright holder, you may use PLOS's Copyright Content Permission form.

Please respond directly to this email or email the journal office and provide any known details concerning your material's license terms and permissions required for reuse, even if you have not yet obtained copyright permissions or are unsure of your material's copyright compatibility.

Potential Copyright Issues:

a. Figures 1, 2: Please confirm whether you drew the images / clip-art within the figure panels by hand. If you did not draw the images, please provide (a) a link to the source of the images or icons and their license / terms of use; or (b) written permission from the copyright holder to publish the images or icons under our CC-BY 4.0 license. Alternatively, you may replace the images with open source alternatives. See these open source resources you may use to replace images / clip-art:

- https://commons.wikimedia.org

- https://openclipart.org/

b. Figure 1: please (a) provide a direct link to the base layer of the map (i.e., the country or region border shape) and ensure this is also included in the figure legend; and (b) provide a link to the terms of use / license information for the base layer image or shapefile. We cannot publish proprietary or copyrighted maps (e.g. Google Maps, Mapquest) and the terms of use for your map base layer must be compatible with our CC-BY 4.0 license.

Note: if you created the map in a software program like R or ArcGIS, please locate and indicate the source of the basemap shapefile onto which data has been plotted.

If your map was obtained from a copyrighted source please amend the figure so that the base map used is from an openly available source. Alternatively, please provide explicit written permission from the copyright holder granting you the right to publish the material under our CC-BY 4.0 license.

Please note that the following CC BY licenses are compatible with PLOS license: CC BY 4.0, CC BY 2.0 and CC BY 3.0, meanwhile such licenses as CC BY-ND 3.0 and others are not compatible due to additional restrictions.

If you are unsure whether you can use a map or not, please do reach out and we will be able to help you. The following websites are good examples of where you can source open access or public domain maps:

* U.S. Geological Survey (USGS) - All maps are in the public domain. (http://www.usgs.gov)

* PlaniGlobe - All maps are published under a Creative Commons license so please cite “PlaniGlobe, http://www.planiglobe.com, CC BY 2.0” in the image credit after the caption. (http://www.planiglobe.com/?lang=enl)

* Natural Earth - All maps are public domain. (http://www.naturalearthdata.com/about/terms-of-use/)

5. Please ensure that your Ethics Statement is available in its entirety at the beginning of your Methods section, under a subheading 'Ethics Statement'. It must include:

i) The name(s) of the Institutional Review Board(s) or Ethics Committee(s)

ii) The approval number(s), or a statement that approval was granted by the named board(s)

iii) (for human participants or donors) - A statement that formal consent was obtained (must state whether verbal/written) OR the reason consent was not obtained (e.g., anonymity).

vi) (for child participants) - Please update your Ethics Statement with confirmation that written informed consent was obtained from the parent/guardian of each participant under 18 years of age.

6. Please provide separate figure files in .tif or .eps format.

For more information about figure files please see our guidelines:

https://journals.plos.org/digitalhealth/s/figures

https://journals.plos.org/digitalhealth/s/figures#loc-file-requirements

7. Please provide an Author Summary. This should appear in your manuscript between the Abstract (if applicable) and the Introduction, and should be 150–200 words long. The aim should be to make your findings accessible to a wide audience that includes both scientists and non-scientists. Sample summaries can be found on our website under Submission Guidelines:

https://journals.plos.org/digitalhealth/s/submission-guidelines#loc-parts-of-a-submission

8. We have noticed that you have uploaded Supporting Information files, but you have not included a list of legends. Please add a full list of legends for your Supporting Information files after the references list.

**Additional Editor Comments (if provided):** **Reviewers' Comments:**

**Comments to the Author**

1. Does this manuscript meet PLOS Digital Health’s publication criteria?

Reviewer #1: Yes

Reviewer #2: Yes

Reviewer #3: Yes

**********

2. Has the statistical analysis been performed appropriately and rigorously?

Reviewer #1: Yes

Reviewer #2: Yes

Reviewer #3: Yes

**********

3. Have the authors made all data underlying the findings in their manuscript fully available (please refer to the Data Availability Statement at the start of the manuscript PDF file)?

The PLOS Data policy

Reviewer #1: Yes

Reviewer #2: Yes

Reviewer #3: No

**********

4. Is the manuscript presented in an intelligible fashion and written in standard English?

Reviewer #1: Yes

Reviewer #2: Yes

Reviewer #3: Yes

**********

**Reviewer #1:**  1. AF was mainly identified by self-report and hospital records. However, AF can be paroxysmal and may be missed. How did the authors assess the possible effect of outcome misclassification on the results?

2. In AlzEye, several retinal markers were significant, but in UK Biobank the most consistent finding was mainly mGCIPL thinning. How do the authors explain this difference between the two cohorts?

3. The models were adjusted for age, sex, ethnicity, hypertension, and diabetes, and in UK Biobank also for smoking and alcohol use. What was the reason for choosing these variables? Were other important AF-related factors, such as BMI, kidney function, blood lipids, medication use, heart failure, or sleep apnea, considered?

4. How did the authors rule out the effect of pre-existing eye diseases that might influence OCT or retinal imaging measurements?

5. If both eyes from one patient were included, or if one patient had repeated records, did the authors make sure that the analysis was separated at the patient level? Please clarify how within-patient correlation was handled.

6. Why was mGCIPL the most consistent signal? Please discuss more fully in the Discussion section why mGCIPL may be related to AF.

7. ECG, wearable devices, and pulse screening (PPG) are already available for AF detection. Compared with these existing methods, what is the added value of retinal imaging for AF screening?

**Reviewer #2:**  This manuscript investigates the association between multimodal retinal imaging parameters and both prevalent and incident atrial fibrillation (AF) using data from two large cohorts (AlzEye and UK Biobank). The study leverages quantitative retinal phenotyping derived from optical coherence tomography and fundus photography. The central finding is an association between thinner macular ganglion cell-inner plexiform layer (mGCIPL) and AF, which was replicated across cohorts.

Overall, this is a strong and timely study at the intersection of ophthalmic imaging and cardiovascular disease. The use of large, well-characterised cohorts and advanced retinal phenotyping is a key strength, and replication across datasets supports the robustness of the findings. However, to strengthen the paper, I recommend some revisions as outlined below. Addressing these issues will improve both the clarity and overall quality of the study.

Major comments:

1. The manuscript would benefit from a more cautious framing of its mechanistic and clinical implications. Several statements appear to extend beyond what can be directly supported by the presented observational data. For example,

• “our report… highlights the mechanistic and potential prognostic utility of retinal imaging in cardiac arrhythmias” (discussion, first paragraph) is an overstatement in the absence of any validation of clinical performance.

• “observed… changes are consistent with our pathomechanistic hypothesis of a causal link to AF” (discussion, fourth paragraph) risk implying a directional causal interpretation that cannot be established from cross-sectional and observational associations alone.

• “these retinal findings are not a substitute for this paradigm, but rather a complementary tool” (discussion, fourth paragraph) overstate translational readiness given that diagnostic accuracy, incremental predictive value, and clinical utility have not been evaluated.

• Implying “early detection,” “risk stratification,” or the ability of retinal imaging to “directly assess end-organ damage” and provide insight into AF pathophysiology also appear premature. (discussion, final paragraph) This is beyond the scope of the current analysis.

• “the novel finding of mINL thinning suggests underlying subclinical ischemia reflecting systemic cardiovascular compromise” introduces a mechanistic interpretation that exceeds what can be directly inferred from the observed association. A more neutral description of mINL thinning as an associated imaging finding, without attributing it to subclinical ischemia or systemic cardiovascular compromise, would better align with the study design.

The authors are encouraged to align the discussion more closely with the observed associations and to avoid extending the interpretation into diagnostic, prognostic, or mechanistic claims that are not directly demonstrated.

2. The adjustment strategy differs between cohorts, with additional lifestyle covariates (smoking and alcohol consumption) included in the UK Biobank models but not in AlzEye. Could the authors clarify the rationale for this discrepancy? If this reflects differences in data availability, it would be helpful to state this explicitly in the Methods section. The authors may want to consider presenting both approaches of adjustment (with and without these additional adjustments in UK Biobank) to aid comparability.

In addition, consideration could be given to other potential confounders such as body mass index, socioeconomic status, medication use, treatment status, and duration of atrial fibrillation, if available. If such variables cannot be incorporated, the potential for residual confounding should be more explicitly acknowledged and discussed as a limitation.

3. The magnitude and clinical significance of the reported associations require clearer contextualisation in the discussion section. While statistically significant, the observed differences in retinal layer thickness (e.g., -1.96 µm in mGCIPL [AlzEye cohort] and -1.19 µm in mGCIPL [UK Biobank cohort]) appear small, and their relevance relative to measurement variability/ inherent noise, age-related changes, and clinical decision-making thresholds is not fully addressed. A more explicit discussion of effect size interpretation would strengthen the translational framing of the work.

4. In the UK Biobank incidence analyses, the relatively short interval between baseline imaging and AF ascertainment may raise the possibility that a proportion of cases had undiagnosed or subclinical AF at baseline. The authors may wish to acknowledge this as a potential limitation. Additionally, the authors could consider performing sensitivity analyses excluding early incident cases (if date of first recorded diagnosis is available), which may help to further clarify the temporal interpretation of the findings.

Minor comments:

1. In the UK Biobank analyses, sensitivity analyses restricting to individuals without prior major adverse cardiovascular events (MACE), as performed in the AlzEye cohort, were not presented. This may reflect the relatively small number of AF cases within UK Biobank after such restriction, limiting statistical power. It would be helpful for the authors to explicitly acknowledge this in the Methods/Results section to clarify why this analysis was not replicated across cohorts.

**Reviewer #3:**  Congratulations to the authors, very interesting work. This report is well structured investigating the association between retinal imaging parameters with prevalent and incident AF. Specific minor comments are provided below:

Minor comments:

1. Caption figure 3: Please be consistent with the abbreviation of ‘AF’

2. Table 1 and 2: Please check missing units in metrics and be consistent ‘n’, ‘N’

3. Caption Table 3: Please can double check typo at the end of the caption: ‘3’

4. Discussion: The term 'high street' imaging is used; perhaps use a different terminology.

5. Methods: Clarify if the retinal measurements were taken from a single eye (and if so, how it was selected) or an average of both eyes.

**********

what does this mean?). If published, this will include your full peer review and any attached files.

**Do you want your identity to be public for this peer review?**  If you choose “no”, your identity will remain anonymous but your review may still be made public.

For information about this choice, including consent withdrawal, please see our Privacy Policy

Reviewer #1: No

Reviewer #2: **Yes:** Krithi Pushpanathan

Reviewer #3: No

**********

**Figure resubmission:**

**Reproducibility:**

To enhance the reproducibility of your results, we recommend that authors of applicable studies deposit laboratory protocols in protocols.io, where a protocol can be assigned its own identifier (DOI) such that it can be cited independently in the future. Additionally, PLOS ONE offers an option to publish peer-reviewed clinical study protocols. Read more information on sharing protocols at https://plos.org/protocols?utm_medium=editorial-email&utm_source=authorletters&utm_campaign=protocols

# Author comment: Author response to Decision Letter 1

**DOI:** 10.1371/journal.pdig.0001661.r002

15 Jun 2026

Attachment Submitted filename: Rebuttal.pdf

# Peer review: Decision Letter 1

**DOI:** 10.1371/journal.pdig.0001661.r003

**Authors:** Feng Liu, Kaisaierjiang Kadier

13 Jul 2026

Response to ReviewersRevised Manuscript with Track ChangesManuscript**Journal Requirements:** **Additional Editor Comments (if provided):** **Reviewers' Comments:**

**Comments to the Author**

Reviewer #1: All comments have been addressed

Reviewer #2: All comments have been addressed

Reviewer #3: All comments have been addressed

**********

publication criteria?

Reviewer #1: Yes

Reviewer #2: Yes

Reviewer #3: Yes

**********

3. Has the statistical analysis been performed appropriately and rigorously?

Reviewer #1: Yes

Reviewer #2: Yes

Reviewer #3: Yes

**********

4. Have the authors made all data underlying the findings in their manuscript fully available (please refer to the Data Availability Statement at the start of the manuscript PDF file)?

The PLOS Data policy

Reviewer #1: Yes

Reviewer #2: Yes

Reviewer #3: Yes

**********

5. Is the manuscript presented in an intelligible fashion and written in standard English?

Reviewer #1: Yes

Reviewer #2: Yes

Reviewer #3: Yes

**********

Reviewer #1: 1. AF was mainly identified by self-report and hospital records. However, paroxysmal AF and asymptomatic AF can easily be missed. Therefore, some participants in the control group may have had undiagnosed AF. This could affect the comparison between AF and non-AF groups. The authors should further discuss in the Discussion section how this outcome misclassification may affect the direction and size of the effect estimates.

2. The models adjusted for age, sex, ethnicity, hypertension, and diabetes, and the UK Biobank models also adjusted for smoking and alcohol use. However, both AF and retinal structure may also be affected by BMI, blood lipids, kidney function, heart failure, sleep apnea, medication use, and socioeconomic status. The authors should clearly state whether these variables were available. If they could not be included, the authors should discuss residual confounding more fully as a limitation.

Reviewer #2: I thank the authors for their comprehensive and thoughtful revision. The manuscript has improved considerably. In particular, the addition of multiple sensitivity analyses (i.e., excluding eye disease and MACE), clarification of the statistical modelling, better contextualisation of effect sizes, and a more nuanced discussion have substantially strengthened the paper.

I only have two minor comments.

1. I appreciate that the authors now acknowledge that approximately 20% of participants developed AF within 24 months of retinal imaging and because AF is typically paroxysmal in its early stages, the findings may be more applicable to prevalent than incident AF. Given this important caveat, the authors may consider softening the concluding statements. One example: the statement that “This study demonstrates that individuals with prevalent and incident AF exhibit thinner mGCIPL and mINL, alongside differences in vascular indices” appears somewhat stronger than the preceding discussion. A wording that better reflects the uncertainty surrounding the incident analyses may improve consistency.

2. ⁠I appreciate that the limitations section now acknowledges the possibility of residual confounding. The authors may wish to expand this slightly by noting that factors such as medication use (particularly anticoagulants), body mass index, chronic kidney disease or kidney function, and AF duration could plausibly influence both retinal morphology and AF status. Explicitly acknowledging these potential sources of residual confounding would further strengthen the discussion.

Reviewer #3: Congratulations to the authors, overall clarity of the work has been improved in this updated version.

**********

what does this mean?). If published, this will include your full peer review and any attached files.

**Do you want your identity to be public for this peer review?**  If you choose “no”, your identity will remain anonymous but your review may still be made public.

For information about this choice, including consent withdrawal, please see our Privacy Policy

Reviewer #1: No

Reviewer #2: No

Reviewer #3: No

**********

**Figure resubmission:**

**Reproducibility:**

To enhance the reproducibility of your results, we recommend that authors of applicable studies deposit laboratory protocols in protocols.io, where a protocol can be assigned its own identifier (DOI) such that it can be cited independently in the future. Additionally, PLOS ONE offers an option to publish peer-reviewed clinical study protocols. Read more information on sharing protocols at https://plos.org/protocols?utm_medium=editorial-email&utm_source=authorletters&utm_campaign=protocols

# Author comment: Author response to Decision Letter 2

**DOI:** 10.1371/journal.pdig.0001661.r004

22 Jul 2026

Attachment Submitted filename: Response to reviewers.pdf

# Editor report: Decision Letter 2

**DOI:** 10.1371/journal.pdig.0001661.r005

**Authors:** Feng Liu, Kaisaierjiang Kadier

27 Jul 2026

Atrial fibrillation and retinal imaging-based oculomics: A cross sectional and longitudinal analysis of two large cohorts

PDIG-D-26-00390R2

Dear Dr Wagner,

We are pleased to inform you that your manuscript 'Atrial fibrillation and retinal imaging-based oculomics: A cross sectional and longitudinal analysis of two large cohorts' has been provisionally accepted for publication in PLOS Digital Health.

Before your manuscript can be formally accepted you will need to complete some formatting changes, which you will receive in a follow-up email from a member of our team.

Please note that your manuscript will not be scheduled for publication until you have made the required changes, so a swift response is appreciated.

IMPORTANT: The editorial review process is now complete. PLOS will only permit corrections to spelling, formatting or significant scientific errors from this point onwards. Requests for major changes, or any which affect the scientific understanding of your work, will cause delays to the publication date of your manuscript.

If your institution or institutions have a press office, please notify them about your upcoming paper to help maximize its impact. If they'll be preparing press materials, please inform our press team as soon as possible -- no later than 48 hours after receiving the formal acceptance. Your manuscript will remain under strict press embargo until 2 pm Eastern Time on the date of publication. For more information, please contact digitalhealth@plos.org.

Thank you again for supporting Open Access publishing; we are looking forward to publishing your work in PLOS Digital Health.

Best regards,

Kaisaierjiang Kadier

Guest Editor

PLOS Digital Health

***********************************************************

**Additional Editor Comments (if provided):**

I think the author has addressed the relevant comments. This study can be accepted.

**Reviewer Comments (if any, and for reference):**
